# Unlocking the Untapped Potential of Endothelial Kinase and Phosphatase Involvement in Sepsis for Drug Treatment Design

**DOI:** 10.3389/fimmu.2022.867625

**Published:** 2022-05-13

**Authors:** Matthijs Luxen, Matijs van Meurs, Grietje Molema

**Affiliations:** ^1^ Department of Pathology and Medical Biology, Medical Biology Section, University Medical Center Groningen, University of Groningen, Groningen, Netherlands; ^2^ Department of Critical Care, University Medical Center Groningen, University of Groningen, Groningen, Netherlands

**Keywords:** endothelial cells (EC), sepsis, sepsis-induced organ injury, sepsis-induced multiple organ failure, drug treatment, signal transduction, kinases and phosphatases

## Abstract

Sepsis is a devastating clinical condition that can lead to multiple organ failure and death. Despite advancements in our understanding of molecular mechanisms underlying sepsis and sepsis-associated multiple organ failure, no effective therapeutic treatment to directly counteract it has yet been established. The endothelium is considered to play an important role in sepsis. This review highlights a number of signal transduction pathways involved in endothelial inflammatory activation and dysregulated endothelial barrier function in response to sepsis conditions. Within these pathways – NF-κB, Rac1/RhoA GTPases, AP-1, APC/S1P, Angpt/Tie2, and VEGF/VEGFR2 – we focus on the role of kinases and phosphatases as potential druggable targets for therapeutic intervention. Animal studies and clinical trials that have been conducted for this purpose are discussed, highlighting reasons why they might not have resulted in the expected outcomes, and which lessons can be learned from this. Lastly, opportunities and challenges that sepsis and sepsis-associated multiple organ failure research are currently facing are presented, including recommendations on improved experimental design to increase the translational power of preclinical research to the clinic.

## Introduction

Sepsis is a complex condition that involves a dysregulated host response to infection. Sepsis is the leading cause of multiple organ failure in patients treated in the intensive care unit, and sepsis-associated mortality is high (1). Our understanding of the underlying processes involved in the onset and progression of sepsis and sepsis-associated multiple organ failure (sepsis-MOF) has increased considerably in the past decades. However, this has not yet resulted in effective drug treatment regimens specifically targeting sepsis-related pathophysiological processes. At present, clinicians merely have infection source control, organ support machines, and antibiotics at their disposal to treat their patients. Therefore, the need for an effective drug treatment for sepsis and sepsis-MOF remains critically high.

The endothelium forms the inner lining of all blood vessels in the body. Since the early 2000s, especially the microvascular endothelium is considered a major player in the pathophysiology of sepsis and sepsis-MOF (2, 3). During sepsis, endothelial cells (EC) are activated by blood-derived stimuli, which can induce microvascular leakage, microvascular inflammation, and vascular hypocontractility, thereby contributing to septic shock and multiple organ failure (4–6). The intracellular signalling pathways that control endothelial activation in response to sepsis are numerous and display a high degree of interconnectedness. This means that different signal transduction pathways involved in the endothelial response to sepsis can share intracellular signalling axes. A corollary to this with regard to therapeutic strategy design is that pharmacological inhibition of one pathway may not be effective, as other pathways are activated unimpededly. Hence, therapeutic targeting of a single intracellular pathway in EC out of a myriad of potential targets is unlikely to improve sepsis outcome.

The aim of this review is to untangle the complexity of six signal transduction pathways that have been described to be involved in EC responses to sepsis conditions, namely NF-κB, Rac1/RhoA GTPases, AP-1, APC/S1P, Angpt/Tie2, and VEGF/VEGFR2^1^. The role of kinases and phosphatases in these pathways will be emphasised throughout this review, as they hold promise as drug targets. The challenges for implementation of kinase inhibitor drug therapy to sepsis patients will also be addressed. The main proteins involved in the activation and downstream signalling of each pathway will be discussed, followed by a description of their engagement in sepsis animal studies, and of clinical trials in sepsis patients that intervene in the pathways of interest. An overview of all pathways will display their interconnectedness and show similarities and differences in the outcomes of their downstream signalling cascades. Discussing the molecular mechanisms of endothelial activation in sepsis in the light of clinical sepsis trials will help explain why some clinical trials failed, while animal studies reported potential benefit. By acknowledging and further mapping this complexity, different subsets of sepsis patients may be identified to define which patient groups may benefit from which treatment.

## Sepsis in the Clinic

Once sepsis-induced hypotension can no longer be counteracted by fluid resuscitation and development of multiple organ failure occurs, the condition has developed into septic shock (1). The combination of both pro- and anti-inflammatory responses observed in sepsis is necessary for clearance of infection and tissue recovery, but an imbalance between both is thought to lead to organ injury and secondary infections (4, 7, 8). A substantial number of factors influence the course of disease progression, including the identity of the invading pathogen, the site of infection, the responses of (micro)vascular beds in organs, influence of comorbidities, and overall heterogeneity between patients (8).

Despite the huge demand for therapies to improve sepsis and sepsis-MOF outcome, and despite decades of research focused on drug development, clinicians currently lack pharmacological treatment options to directly target the processes that contribute to sepsis and sepsis-MOF. Close to 50 million sepsis cases are reported annually worldwide, of which 11 million patients do not survive their ailment (9). Sepsis represents one of the largest burdens to global health, with sepsis-related deaths representing 19.7% of deaths globally (9). Sepsis patients are prone to developing organ failure during the course of their disease, with an estimated 1 in every 3 patients developing acute kidney injury (10). This is associated with an increased likelihood of dying compared to patients suffering from acute kidney injury from other causes (11). Even patients that do survive carry an increased risk of developing chronic kidney disease, hereby also posing a huge socio-economic burden to society (10). In conjunction with the grim prospects for patients suffering from sepsis, this underscores the urgent need for novel and effective treatments for sepsis and sepsis-MOF.

## Endothelial Cell Responses in Sepsis

In sepsis, EC are believed to play a role in many of the occurring pathophysiological processes in response to blood-derived stimuli. Activated EC facilitate leukocyte recruitment and extravasation into the underlying tissue, where they can cause damage to the tissue. In addition, endothelial inflammatory activation induces loss of endothelial barrier function, which can lead to increased microvascular leakage and subsequent tissue hypoxia and organ failure. While under normal conditions these endothelial processes aid clearance of infections and maintenance of homeostasis, exaggerated inflammatory conditions in sepsis overturn the delicate balance between functional and dysfunctional endothelial responses. Dysfunctional EC in sepsis are associated with glycocalyx shedding, decreased organ perfusion and oxygenation, and exacerbated microvascular permeability (2, 12, 13), which indicate an inability to maintain tissue homeostasis. In addition, EC play crucial roles in blood coagulation and thrombus formation, which can become dysregulated during sepsis. Albeit important, altered coagulation during sepsis is not extensively discussed in this review, yet excellent reviews on this topic are available (14, 15).

The various blood vessel segments that constitute the circulatory system - arteries, arterioles, capillaries, post-capillary venules, and veins – all have important roles in response to sepsis that vary from one another based on the type of segment. While modulation of blood flow, blood pressure, and shear stress is primarily controlled *via* the smooth muscle cells in the walls of arteries and smaller arterioles, capillaries and veins contribute little to nothing to these effects. Influx of leukocytes, on the other hand, is especially prominent in post-capillary venules (16), with the exception of lung where leukocyte transmigration primarily takes place in the alveolar capillaries (17). The capillaries and post-capillary venules are of particular interest with respect to sepsis, as they represent the major sites of microvascular leakage (18). Thus, EC from all segments of the circulatory system engage in the response of the body to sepsis, though in each segment they fulfil a different role. Care should be taken regarding the fact that not in all blood vessels nor in all organs similar responses are observed (6).

An integral question in microvascular EC research is if, and to what extent, EC in these different (micro)vascular segments respond to bloodborne stimuli. This depends on the presence of receptors, which may differ between different microvascular segments (19). Furthermore, even if receptors are expressed by EC at similar levels, downstream signalling may differ due to different expression levels of *e.g.* kinases or phosphatases (6, 20), though further research is required to characterise these differential expression patterns in EC *in vivo*. These differences may not only exist between different blood vessel segments, but can also exist between similar segments in different organs, *e.g.* pulmonary capillaries versus renal capillaries (21). Furthermore, variation in expression levels of molecules exists within different microvascular segments of one organ, as illustrated for kinase vascular endothelial growth factor receptor 2 (VEGFR2) in mouse kidney. While in glomeruli, which are specialised capillary networks responsible for filtering the blood, VEGFR2 expression was high, low expression was detected in arterioles and post-capillary venules (22). Organ-specific differences in EC responses to stimuli was illustrated in a study in which mice were exposed to the pro-inflammatory Gram-negative bacterium-derived lipopolysaccharide (LPS). While VEGFR2 expression in kidney had not changed 24 h after LPS administration, VEGFR2 expression in lung significantly decreased already 4 h after LPS administration (23). It is conceivable that the differential expression of receptors and molecules involved in signal transduction by EC in different organs and in different microvascular compartments underlies differential responses of organs in sepsis. By creating a framework of activation status of signal transduction pathways, basal gene/protein expression patterns, and differential changes in gene/protein expression in time in response to sepsis in microvascular compartments in different organs, pathways of interest can be further investigated for their responses to drug intervention studies.

## Signal Transduction Pathways Involved in Endothelial Response to Sepsis

Animal models often used to study the complex pathophysiology of sepsis include endotoxemia following injection with LPS, and cecal ligation and puncture (CLP)-induced polymicrobial sepsis. Endotoxemia results in a high peak of pro-inflammatory cytokines that is resolved relatively rapidly. CLP, on the other hand, is considered the gold standard among sepsis models, as it introduces an active site of bacterial infection in the abdomen. This mimics the course of human sepsis with regards to cytokine response and the development of protracted systemic inflammation more closely (24). LPS is an important component of the inflammatory response following CLP-sepsis, and is systemically elevated as early as 1 h after CLP surgery, though the inflammatory response to both endotoxemia and CLP consists of additional pro-inflammatory stimuli besides LPS (24, 25). Animal responses can be evaluated by *e.g.* pro-inflammatory cytokine levels and markers of endothelial inflammatory activation in the blood, immune cell infiltration into the tissue, circulating markers of organ function and organ damage, and structural integrity of organs assessed by histology.

During the pro-inflammatory phase of sepsis, EC become activated by exogenous agents such as LPS, and by endogenously produced cytokines and chemokines including tumour necrosis factor alpha (TNFα), interleukin (IL)-1β, and IL-6 (24, 26). In EC, LPS is recognised by both Toll-like receptor 4 (TLR4), an important part of the innate immune response against invading pathogens (27), and by retinoic acid-inducible gene I (RIG-I) (28). Immune cells in the host also respond to LPS by producing a plethora of pro-inflammatory cytokines, which can activate EC (29, 30). TNFα is recognised by EC *via* TNFα receptor type 1 (TNFR1) (31), IL-1β *via* a heterodimer of IL-1 receptor 1 and IL-1 receptor accessory protein (32, 33), and IL-6 by binding of IL-6/(soluble) IL-6 receptor complex to gp130 glycoprotein on the endothelial cell surface (34).

EC enter a state of inflammatory activation after stimulation by these pro-inflammatory mediators. This activated state is characterised by increased expression of adhesion molecules including E-selectin and vascular cell adhesion molecule 1 (VCAM-1), which facilitate rolling, adhesion, and transmigration of leukocytes through the endothelium into the underlying tissue. EC also start to produce cytokines and chemokines (22), including IL-6 and monocyte chemoattractant protein 1 (MCP-1), to attract circulating immune cells and signal to other EC (35–39). In addition, Weibel-Palade bodies, which are endothelial granules that contain P-selectin, IL-8, angiopoietin 2 (Angpt2), and von Willebrand Factor (vWF), rapidly release their content into the circulation in response to pro-inflammatory stimuli (40, 41). Endothelial inflammatory activation is also associated with proteolytic cleavage of the extracellular part of transmembrane proteins from the endothelial cell surface, resulting in circulating proteins. An example of this is vascular endothelial cadherin (VE-cadherin), the main structural unit of adherens junctions. Adherens junctions link neighbouring EC and are important contributors to endothelial barrier function, as their structural components serve to prevent free transport of molecules and cells from the blood into the underlying tissue (42, 43). Studies monitoring endothelial dysfunction frequently assess plasma levels of proteins including soluble VE-cadherin (sVE-cadherin), sE-selectin, or vWF to gain insight in the activation status of the endothelium. Similarly, plasma levels of pro-inflammatory cytokines can be monitored in time as determinants of sepsis progression. For instance, elevated levels of Angpt2 and VEGF, which play a role in signal transduction by endothelial-restricted receptor kinases Tie2 and VEGFR2 (discussed in more detail later), have both been associated with more severe disease progression and worsened clinical outcomes in sepsis patients (44, 45).

The following sections will discuss in greater detail six signal transduction pathways that have been described to be involved in EC responses to sepsis *in vivo* or sepsis conditions *in vitro*. Per pathway, the main molecules involved in the signal transduction cascade will be discussed, followed by a description of sepsis animal studies that investigate the pathway. Finally, an overview will be provided of clinical trials in sepsis patients intervening in the described pathways.

### NF-κB Mediates Endothelial Inflammatory Activation in Sepsis

**Figure 1:**
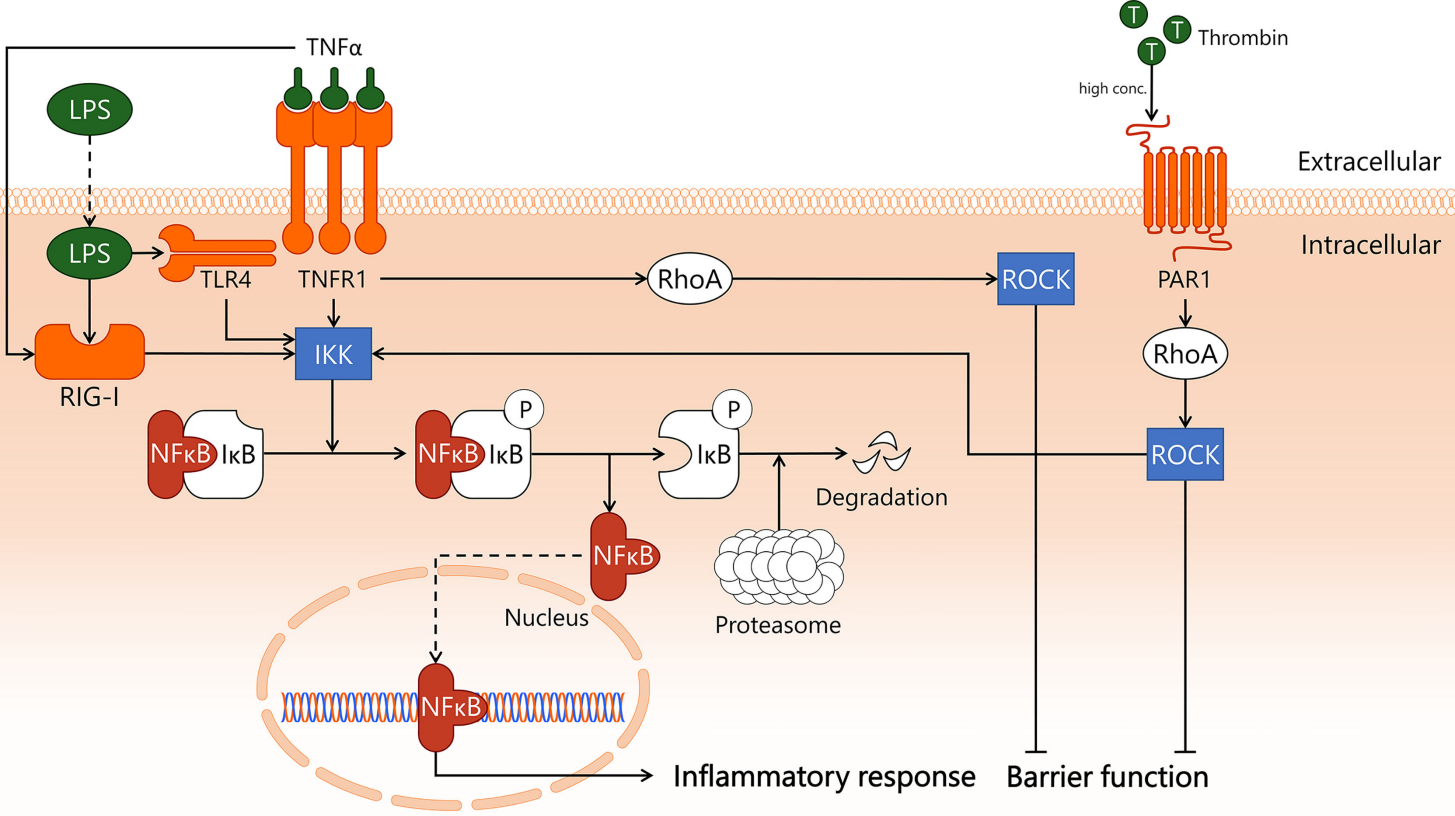
Transcription factor NF-κB induces endothelial inflammation during sepsis. In sepsis, endothelial cells are exposed to pro-inflammatory cytokine TNFα and Gram-negative bacterial product LPS, which incite pro-inflammatory responses. TNFα binds to its receptor TNFR1, activating both IKK and RhoA. TNFα also activates intracellular receptor RIG-I through unknown mechanisms. LPS is taken up by the cell, where it is recognised by both TLR4 and RIG-I, which activate IKK. High levels of thrombin induce PAR1-dependent activation of RhoA, which also activates IKK. In quiescence, IκB masks the nuclear translocation signal of NF-κB, hereby retaining it in the cytosol. Upon activation of IKK, IκB is phosphorylated and dissociates from NF-κB. While IκB is then enzymatically degraded by proteasomes, freed NF-κB translocates to the nucleus to incite an inflammatory response. In addition, the endothelial barrier function is negatively affected, following TNFR1- and PAR1-dependent activation of RhoA. IKK, IκB kinase; IκB, inhibitor of κB; LPS, lipopolysaccharide; NF-κB, nuclear factor kappa-light-chain-enhancer of activated B cells; PAR1, protease-activated receptor 1; RhoA, Ras homolog gene family, member A; RIG-I, retinoic acid-inducible gene I; ROCK, Rho-associated protein kinase; T, thrombin; TLR4, Toll-like receptor 4; TNFR1, tumor necrosis factor alpha receptor 1; TNFα, tumor necrosis factor alpha.

Endothelial inflammatory activation in sepsis can occur *via* recognition of bacterial products such as LPS by TLR4 and RIG-I, or through pro-inflammatory cytokines including TNFα produced by immune cells. TNFα is recognised by EC *via* TNFR1 and TNFα receptor type 2 (TNFR2), though the role of endothelial TNFR2 signalling is incompletely understood (31). Activation of TLR4/RIG-I by LPS as well as activation of TNFR1 by TNFα triggers translocation of NF-κB to the nucleus (31, 46, 47), where it induces transcription of pro-inflammatory genes (48) (**Figure 1**). In the absence of pro-inflammatory stimuli, NF-κB is retained in the cytoplasm *via* interaction with inhibitor of κB (IκB). This interaction conceals the nuclear localisation site of NF-κB, hereby preventing its translocation to the nucleus (49). IκB subtype IκBα can be phosphorylated by IκB kinase (IKK), which is a downstream responder of TNFR1/TLR4/RIG-I (28, 50, 51). This causes IκBα to dissociate from NF-κB, after which it is degraded in proteasomes. The pro-coagulatory protein thrombin also activates IKK in a RhoA-dependent manner (52), which will be discussed in more detail later.

If translocation of NF-κB to the nucleus can somehow be inhibited, a major molecular pathway of pro-inflammatory activation of EC in sepsis is interrupted (53). In mouse CLP-sepsis, treatment with IKK inhibitor IKK 16, a 2-benzamido-pyrimidine, decreased the activity of the IKK complex (54), reduced IκBα phosphorylation, and inhibited nuclear translocation of NF-κB (55). IKK inhibition attenuated sepsis-associated dysfunction of the heart, based on improved cardiac function, and the kidney, based on normalisation of serum creatinine levels (55). Interestingly, IKK inhibition in mouse endotoxemia resulted in a ~30% decrease in lung neutrophil-associated myeloperoxidase activity, whereas in CLP this treatment resulted in a complete abrogation of lung myeloperoxidase activity (55). While no data were provided on which cell types responded to the inhibitor, it would be of interest to expand on this study by localising cells affected by NF-κB blockade, and to distil the role of EC therein.

### Rac1/RhoA GTPases Maintain a Balance Between Protection and Disruption of Endothelial Barrier Function

Integrity of the endothelial barrier is important in homeostasis. In sepsis, disruption of endothelial barrier function leads to microvascular leakage and oedema formation, which is associated with worsened patient survival (43). EC can form intercellular protein-protein connections between adjacent cells, which are crucial in maintaining endothelial barrier function (56). The shape of EC can be altered by changing the properties of the cytoskeleton, thereby affecting cell-cell interactions, which in turn may compromise the integrity of the endothelial barrier. As actin filaments in EC are actively involved in barrier disruptive cytoskeleton rearrangements in sepsis (57, 58), the following section will discuss the signal transduction pathways underlying these processes.

**Figure 2:**
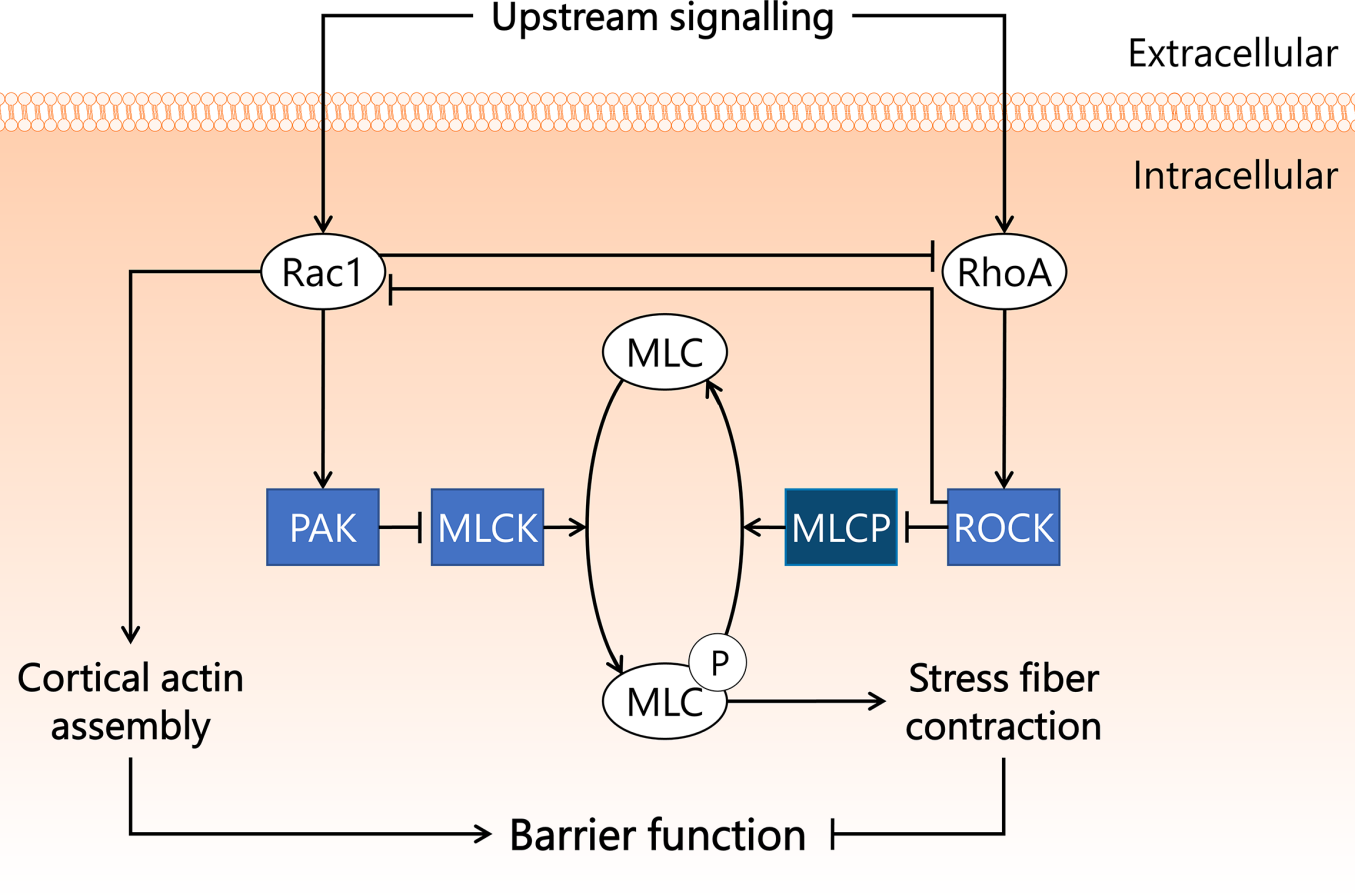
Rac1 and RhoA GTPases exert opposite effects on endothelial barrier function in sepsis. Endothelial barrier integrity is integral for maintaining blood pressure and preventing microvascular leakage and subsequent tissue hypoxia. Rac1 and RhoA GTPases both play important roles in endothelial barrier function, and are activated by a variety of upstream signals associated with sepsis. Rac1 inhibits RhoA and inhibits MLCK in a PAK-dependent manner. RhoA activates its effector kinase ROCK, which inhibits Rac1 activity and phosphorylates MLCP, resulting in inactivation. MLCK induces phosphorylation of MLC, a process reverted by MLCP. Upon phosphorylation, MLC induces the formation and contraction of stress fibres in the cell, leading to reduced cell-cell contacts and dysregulated endothelial barrier function. Activated Rac1 stimulates the assembly of cortical actin, which preserves endothelial barrier function. MLC, myosin light chain; MLCK, myosin light chain kinase; MLCP, myosin light chain phosphatase; PAK, p21-activated kinase; Rac1, Ras-related C3 botulinum toxin substrate 1; RhoA, Ras homolog gene family, member A; ROCK, Rho-associated protein kinase.

Special complexes of actin bundles and myosin called stress fibres are regulated by a small GTPase called Ras homolog gene family, member A (RhoA), most notably *via* activation of Rho-associated protein kinase (Rho kinase), also known as ROCK (59). Stress fibre formation and contraction, as occurs in sepsis conditions, is a result of phosphorylation of the myosin light chain (MLC) (60, 61). The subsequent increase in tension on myosin-associated actin leads to altered cell structure, disrupted endothelial barrier, and increased microvascular permeability (62, 63). The balance between MLC phosphorylation and dephosphorylation is mainly regulated *via* MLC phosphatase and MLC kinase. ROCK reduces MLC phosphatase activity through phosphorylation of MLC phosphatase at an inhibitory site, as a consequence of which MLC dephosphorylation is prevented and stress fibre formation and contraction is stimulated (64, 65). Another small GTPase, Ras-related C3 botulinum toxin substrate 1 or Rac1, has opposing effects on endothelial barrier function compared to RhoA. Rac1 decreases MLC kinase activity through its effector protein p21-activated kinase (PAK) (66), which reduces MLC phosphorylation levels and consequently prohibits stress fibre formation and contraction (67, 68). Rac1 further promotes endothelial barrier function through the assembly of cortical actin, which stabilises the cytoskeleton (69, 70). Rac1 and RhoA also control the activity of one another, with Rac1 inhibiting RhoA (71), and RhoA inhibiting Rac1 *via* ROCK (72), thereby making the molecular balance between protection and disruption of endothelial barrier function even more intricate (**Figure 2**).

Several studies in rodent sepsis models showed involvement of Rac1 and/or RhoA GTPases in the development of sepsis-associated microvascular leakage and inflammation. In a study in which rats were treated with pharmacological ROCK inhibitor Y27632 at 2 mg/kg i.v. directly after CLP instalment, microvascular permeability (measured by FITC-BSA extravasation) decreased ~50% in lung and kidney 8 h after start of CLP compared to untreated rats (73). Another study in rats showed that pre-treatment with Y27632 at 1.5 mg/kg i.p. 20 min prior to CLP ameliorated both sepsis-induced acute lung injury and microvascular leakage in lung (74). Drug administration prior to sepsis onset in these studies does not allow translation of outcomes to therapeutic outcome expectations, as effects of drug pre-treatment are not representative of drug performance during intervention studies. These studies do, however, highlight the involvement of ROCK in the onset and early development of sepsis-induced microvascular permeability in lung and kidney. MicroRNAs (miR) are small non-coding RNAs that can bind to compatible sequences on target mRNAs, thereby sequestering them and preventing translation to protein. One week prior to CLP instalment, mice were injected with miR-539-5p agomir, which mimics the function of endogenous miR-539-5p and suppresses ROCK protein translation. Mice were then sacrificed 24 h after CLP surgery. Treatment with miR-539-5p agomir resulted in reduced pulmonary injury as assessed by histology, and reduced inflammation as assessed by IL-1β and IL-6 mRNA and protein levels in lung (75). The potential applicability of Y27632 and miR-539-5p as therapeutic strategy in sepsis remains to be established, as cell types affected by the treatment, and long-term effects on mortality were not reported. Importantly, ROCK fulfils a key role in maintaining basal endothelial barrier function, as was shown in *in vitro* experiments in human umbilical vein endothelial cells (76). More research is required to establish if in certain microvascular beds ROCK is crucial to maintain basal endothelial barrier function, to circumvent unwanted induction of microvascular leakage and inflammation following systemic ROCK inhibition.

### AP-1 Contributes to Endothelial Inflammatory Activation

Activator protein 1 (AP-1) is a transcription factor heterodimer involved in cell survival and induction of inflammation. The canonical AP-1 complex consists of cellular Fos proto-oncogene (c-Fos) and cellular Jun proto-oncogene (c-Jun), though over 20 members of the AP-1 transcription factor family are known to engage in AP-1 heterodimer formation (77, 78). It is unclear whether particular AP-1 transcription factor family members are cell type- or microvascular compartment-specific, and if so, what the implications are for AP-1-mediated gene transcription (79). The most well-described AP-1 transcription factor family members, c-Fos and c-Jun, can be phosphorylated, which leads to enhanced AP-1-dependent transcription rate of target genes (77, 78). c-Fos is phosphorylated by extracellular signal-regulated kinase (ERK) (80) and p38 mitogen-activated protein kinase (p38) (81), whereas c-Jun is phosphorylated by p38 (82) and c-Jun N-terminal kinase (JNK) (83). These three kinases – ERK, p38, and JNK – are all members of the mitogen-activated protein kinase family that engages in intracellular processes predominantly related to inflammation and cell survival. ERK, p38, and JNK contribute to the transcription and subsequent increase in protein levels of both c-Fos and c-Jun as well, and in this manner are involved in promoting AP-1 transcriptional activity at multiple levels (84–88). Furthermore, p38 has also been reported to lower the activity of both ERK (89) and JNK (87), although the latter interaction has not been confirmed to exist in EC. During endothelial inflammatory activation, the kinases ERK, p38, and JNK become activated by upstream signalling pathways initiated by LPS/TLR4 (90) and TNFα/TNFR1 complexes (91, 92). The PI3K/AKT signalling axis is also activated upon endothelial exposure to LPS (93) and TNFα (94). Furthermore, p38 and JNK are activated by Rac1 and RhoA through mechanisms that are not well described (95–98).

**Figure 3:**
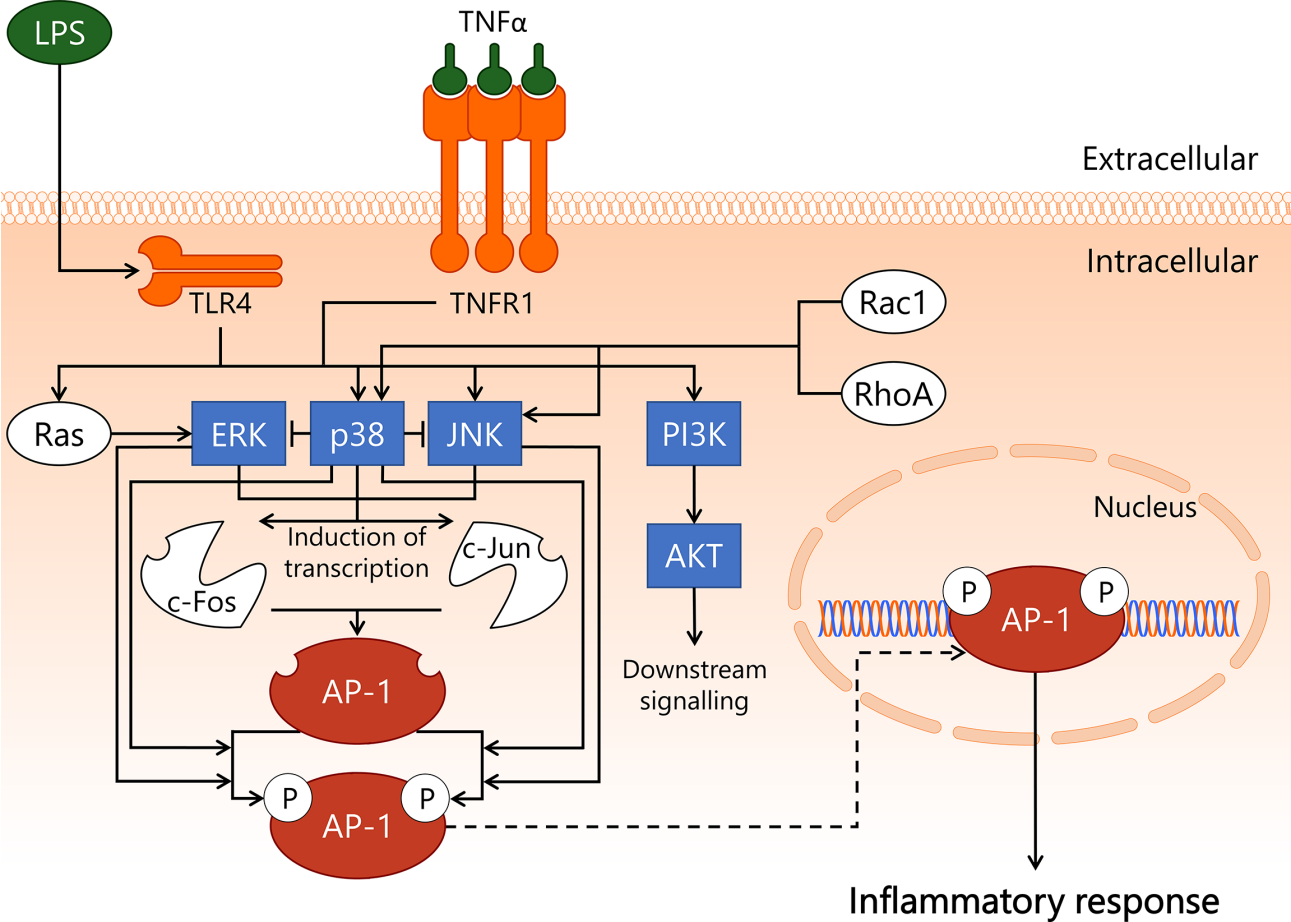
Activation of transcription factor AP-1 in endothelial cells during sepsis. During onset and development of sepsis, transcription factor AP-1 becomes activated in the endothelium. LPS/TLR4 and TNFα/TNFR1 promote activity of PI3K/AKT, Ras/ERK, p38, and JNK kinases, whereas p38 and JNK can also become activated by Rac1 or RhoA. ERK, p38, and JNK induce the transcription of the c-Fos and c-Jun proteins, which can form the AP-1 heterodimer. Phosphorylation of AP-1 enhances its transcriptional function. The c-Fos component of AP-1 is phosphorylated by ERK and p38, whereas the c-Jun component of AP-1 is phosphorylated by p38 and JNK. AP-1 induces transcription of genes mainly associated with inflammation and apoptosis. AKT, protein kinase B; AP-1, activator protein 1; c-Fos, cellular Fos proto-oncogene; c-Jun, cellular Jun proto-oncogene; ERK, extracellular signal-regulated kinase; JNK, c-Jun N-terminal kinase; LPS, lipopolysaccharide; p38, p38 mitogen-activated protein kinase; PI3K, phosphoinositide 3-kinase; Rac1, Ras-related C3 botulinum toxin substrate 1; RhoA, Ras homolog gene family, member A; TLR4, Toll-like receptor 4; TNFR1, tumor necrosis factor alpha receptor 1; TNFα, tumor necrosis factor alpha.

In comparison to NF-κB, AP-1 is less well described in relation to sepsis-related inflammatory activation of EC, and is instead primarily a topic of study in chronic inflammation and oncology (99, 100). As a result, various components of the signal transduction pathway displayed in **Figure 3** represent state-of-the-art knowledge on AP-1 signalling in general, not in EC per se. Since *in vitro* studies in primary EC indicated that inhibition of the NF-κB pathway is insufficient to completely prevent an inflammatory response to *e.g.* LPS, a considerable portion of this response may well be linked to p38-dependent AP-1 activation (101, 102). The importance of AP-1 in sepsis was highlighted in a study using AP-1 decoy oligodeoxynucleotides (ODN), which showed improved survival in mouse CLP-sepsis (103). Furthermore, intervention treatment with the AP-1 decoy ODN 1 h after start of CLP resulted in decreased gene expression of pro-inflammatory cytokines (IL-1β, IL-6, TNFα, MCP-1) in lung and kidney, and a reduction in circulating IL-1β protein levels in blood 18 h after CLP. In addition, treatment with AP-1 decoy ODN induced a significant reduction of neutrophil influx in lung, kidney, and liver 18 h after start of CLP, and reduced sepsis-induced apoptosis in these same three organs (103). Treatment with c-Fos inhibitor T-5224 in mouse endotoxemia improved survival, and resulted in reduced serum blood urea nitrogen and creatinine levels, suggesting improved kidney function (104). Importantly, these studies did not report which cell types were affected by inhibiting AP-1 activity. Hence, the role of AP-1 in EC in sepsis *in vivo* requires further investigation.

A 2001 human endotoxemia study in which healthy volunteers received LPS *via* bolus i.v. injection reported absence of activated JNK in peripheral blood leukocytes, which made the authors conclude that JNK might not be an ideal target when attempting to abrogate inflammation (105). This conclusion does not take a contribution of non-leukocyte cell types to sepsis pathophysiology into account. Strikingly, pre-treatment with pharmacological JNK-inhibitor SP600125 4 h before CLP instalment in rats reduced lung injury (106), with SP600125 administration 1 h after CLP in mice resulting in decreased sepsis-induced lung and liver damage and significantly improved 5-day survival (107).

Also in the early 2000s, multiple studies were performed in which healthy volunteers were closely monitored after being treated with p38 inhibitor drugs followed by systemic LPS challenge. Beneficial effects of p38 inhibition compared to placebo included reduced circulating markers of endothelial inflammatory activation (108), reduced leukocyte activation and attenuated pro-inflammatory cytokine production (109), and maintenance of base level body temperature and heart rate (110). As the effect of p38 inhibition on the endothelium was not reported, the contribution of EC to the reported effects remains unclear. In part due to reported high levels of (hepato)toxicity following p38 inhibitor treatment (111), no follow up has taken place towards a successful therapeutic strategy revolving around p38 inhibition that benefits sepsis patients.

### APC and S1P Receptor Signalling Are Linked and Modulate Endothelial Response in Sepsis

**Figure 4:**
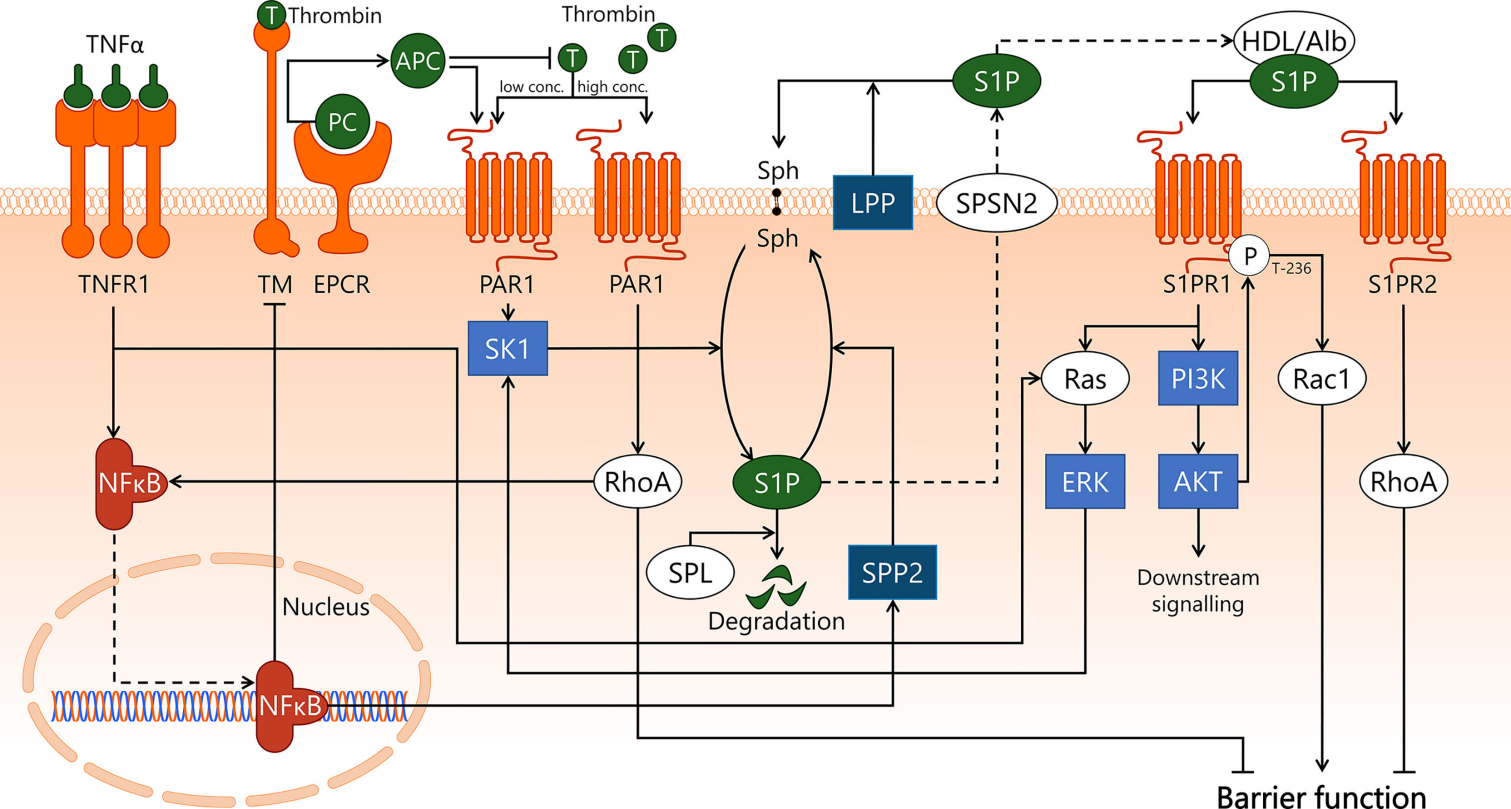
APC/S1P and associated signal transduction pathways in endothelial cells during sepsis. Sepsis pathophysiology is closely associated with microvascular leakage and aberrant coagulation. APC is involved in both processes, by promoting endothelial barrier function and by exerting anti-coagulatory effects. The conversion of protein C to APC by EPCR is stimulated upon association with thrombin/TM, after which APC inhibits thrombin formation. In sepsis-associated inflammation, pro-inflammatory cytokines such as TNFα are recognised by TNFR1 on the endothelial cell surface, leading to activation of Ras/ERK, and activation and subsequent translocation to the nucleus of transcription factor NF-κB. NF-κB induces downregulation of TM, hereby decreasing the turnover of PC to APC. APC and low concentrations of thrombin activate PAR1, which activates SK1. SK1 phosphorylates the lipid sphingosine to form S1P. This process can be reversed by SPP2, whose activity is increased upon NF-κB-induced transcription, and S1P can also be irreversibly degraded by SPL. S1P is transported out of the cell by SPNS2, where it is able to associate with carrier proteins such as albumin and HDL, or can be dephosphorylated by LPP. S1P can then activate S1PR1, leading to activation of Ras/ERK, which stimulates SK1 activity. S1PR1 activation also stimulates PI3K/AKT activity, which enables AKT to phosphorylate S1PR1 at the T-236 site, which is required for its ability to activate Rac1 and promote endothelial barrier function. Alternatively, a disruptive effect on the endothelial barrier function by RhoA is established both by activation of S1PR2 by S1P and through high concentrations of thrombin activating PAR1. In addition, specifically thrombin/PAR1-activated RhoA promotes NF-κB transcriptional activities. AKT, protein kinase B; Alb, albumin; APC, activated protein C; EPCR, endothelial protein C receptor; ERK, extracellular signal-regulated kinase; HDL, high-density lipoprotein; LPP, lipid phosphate phosphatase; NF-κB, nuclear factor kappa-light-chain-enhancer of activated B cells; PAR1, protease-activated receptor 1; PC, protein C; PI3K, phosphoinositide 3-kinase; Rac1, Ras-related C3 botulinum toxin substrate 1; Ras, Ras protein subfamily; RhoA, Ras homolog gene family, member A; S1P, sphingosine-1-phosphate; S1PR1, sphingosine-1-phosphate receptor 1; S1PR2, sphingosine-1-phosphate receptor 2; SK1, sphingosine kinase 1; Sph, sphingosine; SPL, sphingosine-1-phosphate lyase; SPNS2, Spinster homolog 2; SPP2, sphingosine-1-phosphate phosphatase 2; T, thrombin; TM, thrombomodulin; TNFR1, tumor necrosis factor alpha receptor 1; TNFα, tumor necrosis factor alpha.

During sepsis, the balance between coagulation and anti-coagulation is skewed towards heightened coagulation due to increased production of pro-coagulatory factors and decreased production of anti-coagulatory factors, resulting in elevated blood viscosity (112). Sepsis-induced inflammatory activation of EC prompts vWF secretion, which results in microthrombi formation *via* recruitment and activation of platelets (15). Thrombus formation and altered coagulation in sepsis and the role of EC therein are reviewed in detail elsewhere (14, 15). Activated protein C (APC) is an important molecule in sepsis, as it has anti-inflammatory, anti-coagulatory, and anti-thrombotic properties (112, 113). Under homeostatic conditions, relatively high concentrations of APC (5-20 nM) stimulate protease-activated receptor 1 (PAR1) to elicit a protective effect on endothelial barrier function (114, 115). Additionally, activation of PAR1 by pro-coagulatory protein thrombin increases microvascular permeability *via* endothelial RhoA-dependent cytoskeleton rearrangement (116, 117). Hence, stimulation of the PAR1 receptor can have both protective and disruptive effects on endothelial barrier function, depending on its main agonist being either APC or thrombin, respectively (**Figure 4**). In sepsis, the APC:thrombin ratio is commonly decreased due to lowered levels of APC and/or elevated levels of thrombin (14), resulting in increased microvascular leakage following thrombin-dependent PAR1 stimulation (118). PAR1 involvement in human sepsis was investigated in a placebo-controlled study in which healthy volunteers received PAR1 inhibitor vorapaxar 24 h prior to LPS bolus infusion (119). Vorapaxar treatment was associated with reduced activation of coagulation, possibly *via* platelet-expressed PAR1 and decreased endothelial inflammatory activation in response to LPS as assessed by vWF and sE-selectin levels measured in blood. This outcome implies a predominantly harmful effect of PAR1 signalling in endotoxemia, with PAR1 inhibition resulting in reduced endothelial inflammatory activation (119).

Protein C is a zymogen that is primarily produced by the liver, which can be converted to APC by thrombin. In turn, APC inhibits thrombin formation, hereby forming a negative feedback loop that regulates thrombin levels (120). The conversion of protein C to APC is vastly accelerated in the presence of thrombomodulin (TM) and endothelial protein C receptor (EPCR) (121, 122), both of which are expressed on the surface of EC in dedicated microvascular segments (6, 112). The importance of EPCR was emphasised by a study in mouse endotoxemia, in which mice were genetically modified to express less than 10% EPCR protein compared to wildtype controls. Eighteen hours after administration of LPS, mice expressing low levels of EPCR had increased microvascular leakage compared to control mice, particularly in lung, kidney, and brain (123). The authors hypothesised that this outcome may be explained by reduced formation of APC following low EPCR expression, although evidence supporting this claim was not presented. Low APC levels may also be a result of loss of TM from the EC membrane, either due to NF-κB-mediated inhibition of expression or shedding from the endothelial cell surface (124–126). Sepsis-induced endothelial inflammatory activation can result in cleavage of TM from the endothelial membrane, resulting in elevated plasma levels of soluble TM (sTM). sTM is described as a potential endothelial damage marker with prognostic value for disease progression in sepsis (127–129). Interestingly, sTM partially retains its anti-coagulatory and anti-inflammatory functions, among others through continued conversion of protein C into APC and binding of thrombin (113, 130). Reduced expression of TM may be a crucial contributor to decrease APC levels in sepsis, and therefore a key process in sepsis-associated endothelial dysfunction, although further research on sTM is required to better understand its contribution to overall APC production.

#### APC Induces S1PR-Activated Signalling Pathways

A proposed mechanism underlying APC-dependent protection of endothelial barrier function involves the activation of sphingosine kinase 1 (SK1) by APC-dependent PAR1 signalling (114) (**Figure 4**). Intracellular SK1 phosphorylates sphingosine, a constituent of the cell membrane, to convert it into sphingosine-1-phosphate (S1P). S1P is transported out of the cell *via* mechanisms that are not fully understood, although non-ATP dependent transmembrane transporter Spinster homolog 2 is believed to play a role in the export (131). In the blood stream, S1P associates with carriers such as albumin and high-density lipoprotein (132). High-density lipoprotein contains a lipocalin called apolipoprotein M that is not only able to bind S1P, but also delivers it back to the endothelial cell surface to facilitate recognition by sphingosine-1-phosphate receptor 1 (S1PR1) (133). Activation of S1PR1 will lead to downstream signalling *via* the Ras protein subfamily (Ras)/ERK and phosphoinositide 3-kinase/protein kinase B (PI3K/AKT) signalling axes (134, 135). Activated AKT can then phosphorylate S1PR1 on its threonine-236 residue, which will lead to protection of the endothelial barrier function through activation of Rac1 (135). It was shown in EC *in vitro* that after inhibition of SK1 or S1PR1 prior to APC stimulation, this protective effect did not occur (114). This suggests that the protective effect of APC on endothelial barrier function is governed by PAR1, and dependent on both S1P formation and S1P-dependent activation of S1PR1. S1P also activates sphingosine-1-phosphate receptor 2 (S1PR2), which activates RhoA and causes disruption of the endothelial barrier function (136). The effect that will prevail – either S1PR1-induced barrier protection or S1PR2-induced barrier disruption – is believed to depend on expression levels of these S1PRs on the endothelial cell surface (137). Varying expression patterns of S1PRs are reported for different tissues (138), though distribution of microvascular S1PR expression is not well characterised. S1P availability is also dependent on its turnover rate, which occurs intracellularly either *via* cleavage by S1P lyases (139) or dephosphorylation by S1P phosphatases (140), or extracellularly *via* dephosphorylation by lipid phosphate phosphatases (141, 142). In human umbilical vein endothelial cells exposed to TNFα for 4 h, sphingosine-1-phosphate phosphatase 2 expression was induced up to 400 times, and its activity was increased (143). This illustrates involvement of pro-inflammatory signalling pathways in S1P turnover.

Various animal studies investigated the involvement of S1P and S1PRs in the onset and progression of sepsis. The 2-day and 10-day survival of S1PR2 KO mice was studied after intraperitoneal injection of *E. coli* (144) respectively induction of polymicrobial sepsis by CLP (145). Both studies found a significant increase in survival of S1PR2 KO mice compared to WT, and primarily attributed this finding to altered macrophage behaviour. Even though S1PR2 is abundantly expressed by macrophages, effects of genetic deletion on the behaviour of other cell types that also express S1PR2 – including EC – were not addressed. Treatment of mice with general S1PR agonist FTY720 and S1P lyase inhibitor 4-deoxypyridoxine before inducing sepsis by peritoneal contamination and infection (PCI) reduced microvascular leakage in lung and liver, and dampened IL-6, TNFα, and MCP-1 production. This indicates a positive effect of increased S1PR activation and elevated availability of S1P on sepsis-associated microvascular integrity loss and immunological response (146). It is difficult to identify the exact molecular mechanisms responsible for these outcomes, as all S1PRs are likely affected by this treatment.

Successful outcomes following treatment with S1P-system modifying drugs in animal sepsis models are not universal, as a study utilising a rat sepsis model called colon ascendens stent peritonitis (CASP) reported that treatment with S1PR1-agonist SEW2871 at 0.5 mg/kg i.v. 12 h after induction of sepsis did not improve microvascular leakage in mesenteric venules or serum creatinine levels in these animals. On the contrary, SEW2871 treatment resulted in bradyarrhythmia and cardiac arrest, which exacerbated mortality compared to untreated rats. The molecular mechanisms underlying these symptoms are speculated to be a result of S1PR1 internalisation in response to prolonged exposure to SEW2871 (147). In mouse CLP-sepsis, on the other hand, SEW2871 administration 6 h after start of CLP at 10 mg/kg i.p. exerted a protective effect on kidney function, as measured by reduced blood urea nitrogen and creatinine protein levels in serum, and kidney morphology 18 h after CLP (148). The differences in outcome may be explained by differences between the pathophysiology of CASP and CLP sepsis, which may affect the pharmacological effect of SEW2871. Furthermore, the timing and dosage between both studies also differed. SEW2871 administration at 0.5 mg/kg 12 h after sepsis induction failed to improve sepsis-induced leakage or kidney function, while 10 mg/kg 6 h after sepsis did improve kidney function. These results could indicate that S1PR1 is involved in early onset of sepsis, higher dosages of S1PR1 agonist are required to invoke protective effects, and that there is a limited window of treatment after sepsis onset for SEW2871 to attenuate sepsis-induced organ dysfunction.

### Protective Signalling of Tie2 in EC Is Disrupted in Sepsis

**Figure 5:**
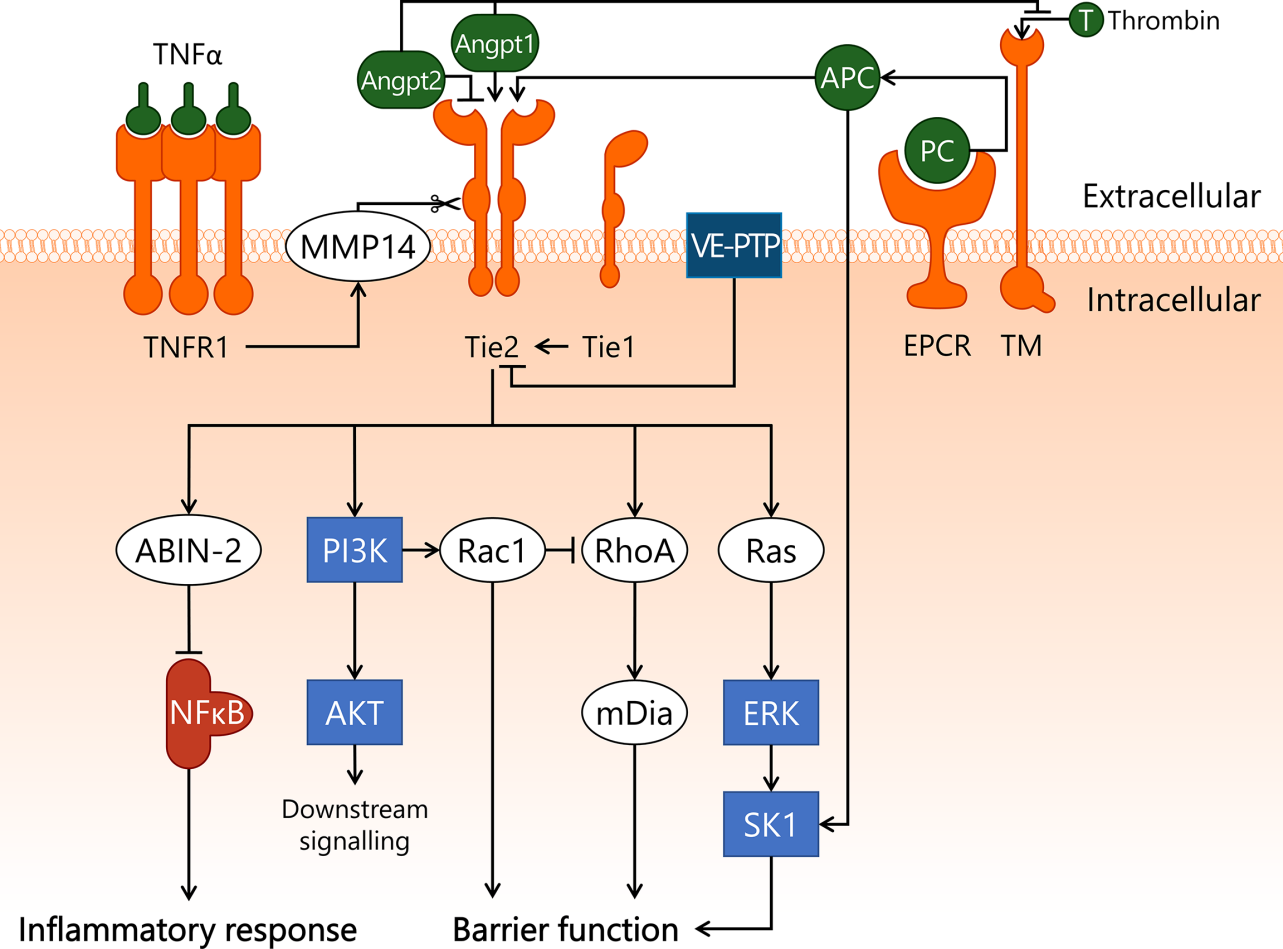
Endothelial Angpt/Tie2 signalling is compromised in sepsis. A key regulator of endothelial homeostasis – Tie2 – is heavily modulated in sepsis, which can result in endothelial dysfunction. Angpt1 and APC serve as agonists for Tie2, whereas Angpt2 competitively binds to Tie2, preventing its activation. Angpt1 and Angpt2 can also bind to TM, hereby inhibiting EPCR/TM/thrombin-dependent conversion of PC to APC. Tie2 activity is further modulated through association with Tie1, which lowers the activation threshold of Tie2 to promote its activation, and VE-PTP, which decreases Tie2 activation status through dephosphorylation. TNFα/TNRF1 promotes cleavage of the extracellular domain of Tie2 via MMP14. Upon activation, Tie2 exerts an anti-inflammatory effect by activating ABIN-2, which prevents nuclear translocation of NF-κB. In addition, Tie2 activates Ras/ERK/SK1 and PI3K/AKT. Finally, Tie2 has a protective effect on endothelial barrier function through PI3K-dependent activation of Rac1, and RhoA-dependent activation of mDia. ABIN-2, A20-binding inhibitor of NF-κB activation-2; AKT, protein kinase B; Angpt1, angiopoietin-1; Angpt2, angiopoietin-2; APC, activated protein C; ERK, extracellular signal-regulated kinase; mDia, mammalian Diaphanous-related formin Dia; MMP14, matrix metalloproteinase-14; NF-κB, nuclear factor kappa-light-chain-enhancer of activated B cells; PI3K, phosphoinositide 3-kinase; Rac1, Ras-related C3 botulinum toxin substrate 1; Ras, Ras protein subfamily; RhoA, Ras homolog gene family, member A; SK1, sphingosine kinase 1; T, thrombin; Tie1, tunica intima endothelial kinase 1; Tie2, tunica intima endothelial kinase 2; TM, thrombomodulin; TNFR1, tumor necrosis factor alpha receptor 1; TNFα, tumor necrosis factor alpha; VE-PTP, vascular endothelial protein tyrosine phosphatase.

Another endothelial protein that the sepsis field has turned its attention to, is receptor kinase tunica intima endothelial kinase 2 (Tek), also known as Tie2. Tie2 exhibits enriched endothelial expression, but is also present in a variety of other cell types (149). Two ligands play a key role in regulating Tie2 signalling. Under non-inflammatory conditions, binding of the agonist angiopoietin 1 (Angpt1) to Tie2 induces anti-inflammatory (150) and barrier protective effects (151, 152) (**Figure 5**). The second ligand is the antagonist Angpt2, which competitively binds to Tie2, thus inhibiting Angpt1-dependent protective signal transduction in EC (153). Activation of Tie2 leads to its internalisation and degradation, thereby reducing Tie2 availability at the cell surface (154). APC is also reported to induce Tie2 downstream signalling through functioning as an agonist for Tie2, and treatment with APC 5 min prior to LPS administration prevented LPS-induced microvascular leakage in lung and kidney in a Tie2-dependent manner (155). Interestingly, TM-facilitated formation of APC is inhibited by binding of both Angpt1 and Angpt2 to TM (156), suggesting involvement of angiopoietins in coagulation, and adding an additional connection between the Tie2 pathway and APC.

The ability of Tie2 to initiate downstream signalling in response to its agonists is controlled by interactions with several associated regulatory proteins. Tie2 interacts with orphan receptor Tie1, which lowers the threshold of Tie2 activation under resting conditions (157, 158). This lowered activation threshold of Tie2 enables Angpt2 to function as a Tie2 agonist instead (157, 159), though the extent to which this interaction occurs in microvascular beds has not been reported. During inflammatory conditions Tie1 is cleaved, thereby reverting the lowered Tie2 activation threshold (157). Tie2 itself is also cleaved by matrix metalloproteinase-14 in response to inflammatory mediators such as TNFα, thereby inhibiting downstream signalling of Tie2 (160). Finally, Tie2 can be dephosphorylated by membrane-bound vascular endothelial protein tyrosine phosphatase (VE-PTP), leading to reduced Tie2-mediated signal transduction (161).

Downstream signalling of activated Tie2 includes multiple pathways and cellular processes. A Tie2-dependent anti-inflammatory effect is exerted through stimulation of A20-binding inhibitor of NF-κB activation-2 (ABIN-2) (150). ABIN-2 inhibits IKK, thereby preventing NF-κB translocation to the nucleus and interfering with inflammatory gene transcription (162). Tie2 also activates the PI3K/AKT pathway, after which PI3K stimulates Rac1. Rac1 then inhibits RhoA, resulting in protection of endothelial barrier function (71). Tie2 also activates mammalian Diaphanous-related formin Dia (mDia) in a RhoA-dependent manner (151). mDia exerts a protective effect on endothelial barrier function *via* an inhibitory interaction with the VEGFR2 pathway that will be discussed in more detail below. Finally, activation of Tie2 induces signalling through the Ras/ERK pathway (163), leading to activation of SK1 (164), thereby providing another connection between the Angpt/Tie2 pathway and the APC/S1P pathway.

Angpt/Tie2 signalling is disrupted during sepsis, due to elevated levels of Angpt2 (165). This results in increased endothelial inflammation and microvascular leakage (45, 166). Furthermore, in mouse endotoxemia Tie2 is downregulated in several organs, including brain, kidney and liver (167, 168). A study in renal biopsies of sepsis patients with renal dysfunction showed that mRNA levels of Tie2 were significantly decreased in renal biopsies of sepsis patients (169), though this effect was not reported in other studies (23, 170). Moreover, mRNA expression of both Angpt1 and Angpt2 were decreased in the kidney of sepsis patients (169). This indicates that therapeutic intervention in this pathway will require an approach that goes beyond merely altering the systemic Angpt1:Angpt2 ratio. Indeed, Angpt2 knockout failed to prevent kidney dysfunction in mouse endotoxemia, although a decrease in endothelial inflammatory activation was observed (168).

Angpt2-binding and Tie2-activating antibody (ABTAA), which induces oligomerisation of Angpt2 and subsequently activates Tie2, was used as intervention treatment in sepsis-associated microvascular dysfunction (171). In three different mouse sepsis models - CLP-sepsis, LPS endotoxemia, and *S. aureus* injection – ABTAA treatment 6 h and 18 h after sepsis induction improved survival rate. ABTAA was shown to reduce microvascular leakage in the lung assessed at 30 h after LPS injection. Furthermore, upon ABTAA administration 6 h after start of CLP, reduced heparanase protein expression was observed in lung and in glomeruli in the kidney 12 h post-CLP, indicating improved endothelial glycocalyx integrity. Finally, administration of ABTAA 6 h after the start of CLP lowered TNFα, IL-6, and Angpt2 protein levels in serum at 12 h, 18 h, and 24 h post-CLP (171). These anti-inflammatory capabilities of increased Tie2 activation likely contribute to normalisation of EC behaviour in sepsis. The above findings indicate that Tie2 signalling plays an important role in the onset and progression of various pathophysiological processes taking place during sepsis. Crucially, more research is required to elucidate which organs and which microvascular compartments are affected by altered Tie2 signalling during sepsis, before suitable targets within the Tie2 pathway can be identified for novel therapeutic strategies.

### VEGFR2 Is Involved in EC Barrier Disruption in Sepsis

VEGF/VEGFR2 is a molecular signalling axis that controls microvascular permeability, by modifying endothelial behaviour in sepsis. Elevated plasma levels of VEGF have been reported in sepsis patients (172), which may lead to increased VEGF-dependent activation of VEGFR2. In mouse CLP-sepsis, treatment with anti-VEGF neutralising antibody bevacizumab at 0.1 mg/kg i.p. 1 h before CLP or 6 h after CLP improved survival rate (173), though these findings could not be reproduced in a later study in which bevacizumab was administered at 0.5 mg/kg i.p. immediately prior to CLP (174). Pre-treatment with bevacizumab 1 h before LPS injection i.p. in mice reduced microvascular permeability in kidney, lung, and spleen at 24 h after LPS administration (173). Pre-treatment with a neutralising antibody targeting both Angpt2 and VEGF (A2V) in mouse CLP-sepsis alleviated endothelial inflammatory activation in kidney as assessed by reduced intercellular adhesion molecule 1 (ICAM-1) protein expression in peritubular capillaries, reduced pulmonary permeability, and improved 5-day survival (175). These findings suggest that decreased interaction of VEGF and Angpt2 with their respective receptors results in survival benefit in this particular setting. However, it was not reported whether VEGF and Angpt2 plasma levels or VEGFR2 and Tie2 activation levels were affected in response to A2V treatment, which calls for further studies into the underlying molecular mechanisms of this treatment.

**Figure 6:**
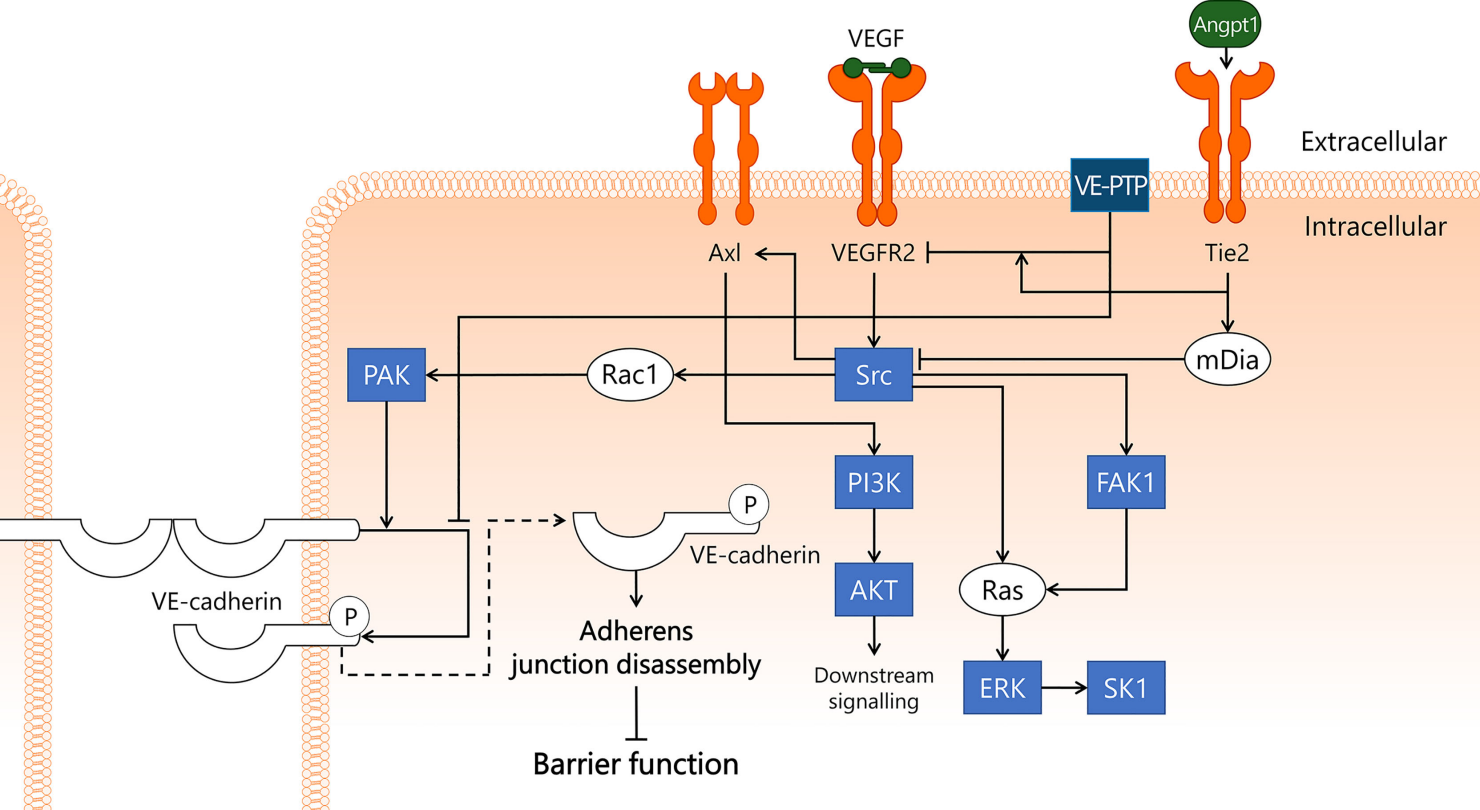
VEGF/VEGFR2 pathway disrupts endothelial barrier function in sepsis. VEGFR2 is well-known for its role in angiogenesis, but it is also thought to play a key role in sepsis-induced endothelial barrier dysfunction. VEGF binds to VEGFR2, leading to activation of Src kinase. Src then binds to and activates FAK1, which induces activation of Ras/ERK/SK1 through both Src and FAK1. In addition, Src activates PI3K/AKT via Axl, and Rac1/PAK. PAK phosphorylates VE-cadherin, leading to its internalisation and subsequent disassembly of adherens junctions, which results in disrupted endothelial barrier function. This process is inhibited by VE-PTP, which closely associates with VE-cadherin, hereby preventing its phosphorylation. VE-PTP also dephosphorylates VEGFR2, a process that is promoted by the vicinity of Tie2. Tie2 activation induces activation of mDia, which sequesters Src and prevents its involvement in the VEGF/VEGFR2 pathway. AKT, protein kinase B; Angpt1, angiopoietin-1; Axl, Axl receptor tyrosine kinase; ERK, extracellular signal-regulated kinase; FAK1, focal adhesion kinase 1; mDia, mammalian Diaphanous-related formin Dia; PAK, p21-activated kinase; PI3K, phosphoinositide 3-kinase; Rac1, Ras-related C3 botulinum toxin substrate 1; Ras, Ras protein subfamily; SK1, sphingosine kinase 1; Src, Src protein tyrosine kinase; Tie2, tunica intima endothelial kinase 2; VEGF, vascular endothelial growth factor; VEGFR2, vascular endothelial growth factor receptor 2; VE-cadherin, vascular endothelial cadherin; VE-PTP, vascular endothelial protein tyrosine phosphatase.

Upon binding of VEGF, VEGFR2 will autophosphorylate several tyrosine sites in its intracellular domain to initiate downstream signalling *via* Src protein tyrosine kinase (Src) (176, 177) (**Figure 6**). VE-PTP can dephosphorylate these autophosphorylation sites, hereby reducing the ability of VEGFR2 to activate its downstream signalling targets (178). VE-PTP-induced dephosphorylation of VEGFR2 is dependent on Tie2 (178). In addition, Tie2 stimulates mDia to sequester Src (151), rendering Src unable to induce further downstream VEGFR2 signal transduction. Activated Src interacts with focal adhesion kinase 1 (FAK1), resulting in phosphorylation and increased activity of both Src and FAK1 (179). Both Src and FAK1 then activate the Ras/ERK signalling pathway (180). FAK1 is most well-known for its role in integrin signalling, and is implicated in endothelial inflammatory activation (181–183). In rat endotoxemia, knockdown of FAK1 by siRNA elicited a protective effect through a reduction of injurious inflammation-induced remodelling of lung tissue (184). FAK1 knockdown also inhibited LPS-induced inflammatory activation in human umbilical vein endothelial cells (183), as well as prevented injurious remodelling of cardiac tissue in rat endotoxemia (185). These studies underscore the role of FAK1 in some deleterious processes following endotoxemia-induced inflammation, yet much remains to be elucidated regarding the exact molecular involvement of FAK1 in inflammation in general and in sepsis in particular. Importantly, further identification of the cell types in which FAK1 contributes to these processes is required before therapeutic strategies and accompanying biomarker readouts can be designed.

In addition to FAK1, Src activates transmembrane Axl receptor tyrosine kinase (Axl), which then leads to activation of the PI3K/AKT pathway (186). Axl can also be activated by its extracellular ligand, growth arrest specific 6 (Gas6), which is increased in sepsis in the blood and is suggested to play a role in systemic inflammation (187). A clinical study in sepsis patients reported that elevated Gas6 levels were associated with increased mortality (188). At this point, the exact role of Gas6 in sepsis is not well understood, nor is it known whether interactions with Axl in microvascular EC contribute to morbidity and/or mortality in sepsis patients.

Finally, Src plays a crucial role in VEGFR2-induced endothelial barrier dysfunction in conjunction with VE-cadherin, the main structural component of adherens junctions that is responsible for the physical interaction between neighbouring EC. Src induces activation of Rac1 and its effector protein PAK, which then phosphorylates VE-cadherin (176). This phosphorylation leads to internalisation of VE-cadherin, resulting in disassembly of the adherens junctions, loss of endothelial barrier function, and ultimately increased microvascular leakage (176, 189). In mice, stable VEGFR2/VE-cadherin complex formation in heart EC was disrupted in a Src-dependent fashion following VEGF injection (190). Internalisation of phosphorylated VE-cadherin may be prevented by dephosphorylation by VE-PTP (191). In this manner, VE-PTP contributes to adherens junction stability and endothelial barrier integrity. In addition to being internalised after phosphorylation, VE-cadherin can also be shed from the endothelial cell surface into the circulation. A study in sepsis patients reported elevated sVE-cadherin levels in a subset of patients that were suffering from acute kidney injury (192). There are also organ-specific variations in VE-cadherin expression in response to inflammatory stimuli, as VE-cadherin mRNA levels in the lung decreased significantly 4 h after i.p. LPS administration, whereas no changes were observed in the kidney (23). It remains to be determined whether loss of VE-cadherin in sepsis is occurring from the majority of EC, or only affects specific microvascular compartments, and whether sVE-cadherin exerts a signalling function through recognition by and interaction with EC. By quantifying VE-cadherin protein levels in different microvascular beds in organs during onset and development of sepsis to determine changes in expression levels in response to sepsis, or by studying the effects of exposing EC to sVE-cadherin, a deeper understanding of the role of VE-cadherin in sepsis can be obtained.

### Clinical Sepsis Trials Intervening in the Highlighted Signal Transduction Pathways

Several clinical trials in severe sepsis patients have been conducted to assess the effects of intervening in one of the discussed signal transduction pathways. Studied molecular targets include components of the NF-κB pathway (**Figure 1**). For example, blockade of TLR4 was employed in a clinical trial in which sepsis patients were treated with eritoran, a synthetic TLR4 antagonist that reduced LPS-dependent activation of TLR4. No effects on 28-day mortality were observed compared to patients who received placebo (193). TAK-242, also known as resatorvid, is a small molecule inhibitor of TLR4 that interacts with the intracellular domain of TLR4, thereby inhibiting the downstream signalling cascade initiated by bacterial LPS. TAK-242 treatment in sepsis patients did not affect plasma IL-6 levels, indicating no overall change in inflammatory status of the patients. Importantly, 28-day mortality was also not improved after drug treatment (194).

Another approach investigated blockade of TNFα to blunt the inflammatory response in sepsis patients. As a primary mediator of systemic inflammation in sepsis, its neutralisation could in theory prevent further downstream activation of harmful responses, as shown in baboons (195). This rationale was followed in a study in which a neutralising polyclonal anti-TNFα antibody fragment called AZD9773 was administered to patients with severe sepsis. AZD9773 effectively reduced circulating TNFα plasma levels, yet did not result in reduction of IL-6 plasma levels, nor did it improve 90-day survival of patients (196).

Taking out one major upstream component of an inflammatory signalling pathway to counteract the detrimental effects of sepsis on the microvasculature seems a straightforward manner to modulate microvascular inflammation and leakage. However, the clinical trials discussed above illustrate that this approach is not by definition effective in treating sepsis patients. This may be explained by the existence of compensatory molecular mechanisms, where alternative signal transduction pathways become activated to compensate for the lack of activation of the inhibited pathway. For instance, exposure of EC to LPS or TNFα both lead to activation and translocation of NF-κB. When TNFα-dependent activation of NF-κB is inhibited in sepsis patients, it is conceivable that this is insufficient to abolish inflammatory activation, as *e.g.* LPS-dependent endothelial inflammatory activation will still be possible. Similarly, when TLR4 is blocked with drugs like eritoran or TAK-242, the activation of RIG-I by LPS may remain unaffected or in theory could even increase to compensate for reduced TLR4 activation.

A major setback to the field of sepsis and sepsis-MOF research was the PROWESS-SHOCK clinical trial published in 2001. The overall aim was to improve sepsis patient outcome through administration of APC, which was hypothesised to prohibit sepsis-related coagulopathy and attenuate inflammatory activation. Sepsis patients were treated with recombinant human APC, also known as drotrecogin alfa (activated) (DrotAA, or Xigris). DrotAA was initially reported to reduce mortality in patients with severe sepsis (197), but in a later study no such evidence was found. Moreover, DrotAA caused bleeding complications due to its anti-coagulatory effects (198). The outcome of the PROWESS-SHOCK study has played a role in perpetuating the stigma surrounding sepsis research as “the graveyard for pharmaceutical companies” (199). This is a self-fulfilling prophecy, for a condition as complex and heterogeneous as sepsis will require large clinical studies with proper patient subtyping to establish treatment effectiveness, to increase our understanding of sepsis pathophysiology, and to identify biomarkers for different disease stages. When pharmaceutical companies are hesitant to invest in new sepsis research because it is deemed too high-risk, the progress of the sepsis research field as a whole will consequently be slowed down considerably.

The overall complexity and heterogeneous presentation of sepsis led to the identification of 4 distinct clinical phenotypes, including a phenotype characterised by abnormal coagulation (200). The SCARLET clinical trial investigated whether the anti-coagulatory capabilities of sTM (a.k.a. ART-123) could ameliorate the disseminated coagulation in patients suffering from sepsis-associated coagulopathy. However, treatment with recombinant human sTM did not reduce 28-day mortality (201). A follow-up clinical trial called SCARLET2 was later launched, focusing on severe sepsis patients with coagulopathy and dysfunction of at least one organ, but the study was withdrawn to accommodate changes in study design based on new results (NCT03517501).

While anti-VEGF neutralising antibody bevacizumab showed variable results in mouse sepsis (173, 174), a clinical trial was set up to test its effectiveness in sepsis patients, in particular in preventing development of sepsis-associated acute respiratory distress syndrome. This study was prematurely withdrawn before participants were enrolled (NCT01314066).

The above discussed clinical trials include interventions in the NF-κB, APC/S1P, and VEGF/VEGFR2 signalling pathways. No studies intervening in the AP-1, Rac1/RhoA GTPases, and Angpt/Tie2 signalling pathways have yet reached the stage of clinical trials. These pathways still represent promising therapeutic targets to ameliorate endothelial dysfunction in sepsis. Furthermore, it is important to note that this review focuses on the role of the endothelium in sepsis yet the discussed signalling pathways are not exclusive to EC. As a result, clinical trials intervening in these pathways likely affect the endothelium, but also a wide range of other cell types. For this reason, it is vital that markers for endothelial damage and/or inflammation are being investigated and reported, as it is one of few available tools to study EC responses in patients. In actuality, endothelial damage and inflammation markers are seldomly studied or reported in clinical trials, thereby obscuring the effects of these treatments on the endothelium.

## Endothelial-Based Anti-Sepsis Therapeutics – A Blueprint

### Kinase and Phosphatase Inhibitor Drugs for Sepsis Treatment: Opportunities and Challenges

**Figure 7:**
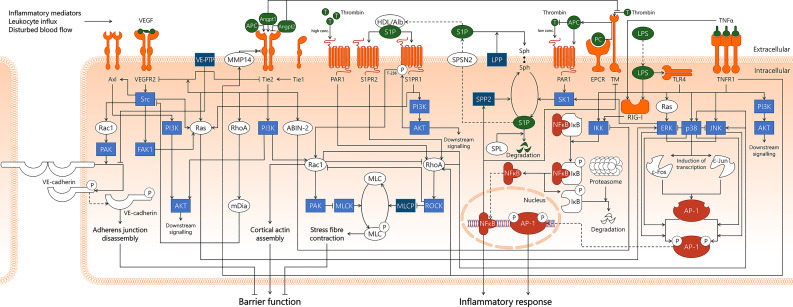
Integration of signal transduction pathways involved in endothelial responses in sepsis that affect endothelial barrier function and inflammatory responses. From left to right, this figure displays the VEGF/VEGFR2, Angpt/Tie2, Rac1/RhoA GTPases, APC/S1P, NF-κB, and AP-1 signal transduction pathways. This overview illustrates the complexity of the interactions of pathways involved in the endothelial response to sepsis, as well as displaying the numerous links that exist between different pathways. Coagulation-affecting pathways are not separately indicated for clarity reasons. ABIN-2, A20-binding inhibitor of NF-κB activation-2; AKT, protein kinase B; Alb, albumin; Angpt1, angiopoietin-1; Angpt2, angiopoietin-2; APC, activated protein C; AP-1, activator protein 1; Axl, Axl receptor tyrosine kinase; c-Fos, cellular Fos proto-oncogene; c-Jun, cellular Jun proto-oncogene; EPCR, endothelial protein C receptor; ERK, extracellular signal-regulated kinase; FAK1, focal adhesion kinase 1; HDL, high-density lipoprotein; IKK, IκB kinase; IκB, inhibitor of κB; JNK, c-Jun N-terminal kinase; LPP, lipid phosphate phosphatase; LPS, lipopolysaccharide; mDia, mammalian Diaphanous-related formin Dia; MLC, myosin light chain; MLCK, myosin light chain kinase; MLCP, myosin light chain phosphatase; MMP14, matrix metalloproteinase-14; NF-κB, nuclear factor kappa-light-chain-enhancer of activated B cells; p38, p38 mitogen-activated protein kinase; PAK, p21-activated kinase; PAR1, protease-activated receptor 1; PC, protein C; PI3K, phosphoinositide 3-kinase; Rac1, Ras-related C3 botulinum toxin substrate 1; Ras, Ras protein subfamily; RhoA, Ras homolog gene family, member A; RIG-I, retinoic acid-inducible gene I; ROCK, Rho-associated protein kinase; S1P, sphingosine-1-phosphate; S1PR1, sphingosine-1-phosphate receptor 1; S1PR2, sphingosine-1-phosphate receptor 2; SK1, sphingosine kinase 1; Sph, sphingosine; SPL, sphingosine-1-phosphate lyase; SPNS2, Spinster homolog 2; SPP2, sphingosine-1-phosphate phosphatase 2; Src, Src protein tyrosine kinase; T, thrombin; Tie1, tunica intima endothelial kinase 1; Tie2, tunica intima endothelial kinase 2; TLR4, Toll-like receptor 4; TM, thrombomodulin; TNFR1, tumor necrosis factor alpha receptor 1; TNFα, tumor necrosis factor alpha; VEGF, vascular endothelial growth factor; VEGFR2, vascular endothelial growth factor receptor 2; VE-cadherin, vascular endothelial cadherin; VE-PTP, vascular endothelial protein tyrosine phosphatase.

The collective effort of *in vitro* studies, animal experiments, analyses of human samples obtained from patients, and clinical trials conducted in sepsis patients have contributed tremendously to our understanding of the cellular and molecular pathways in EC that contribute to the onset and progression of sepsis and sepsis-MOF. This review described six signal transduction pathways involved in endothelial activation in sepsis and in conditions mimicking sepsis, that play crucial roles in modulating coagulation, microvascular permeability, and leukocyte recruitment. An integrated schematic presentation of what we know so far about these pathways is represented in **Figure 7** (relevant references are summarised in **Supplementary Table 1**), in which the sheer number of connections both within and between signal transduction pathways can be appreciated. We now face the challenge of not only expanding this knowledge further, but also to translate it into effective drug treatment strategies. In the clinical trials discussed, the underlying rationale often revolves around neutralisation of systemic pro-inflammatory mediators. Therapeutic regimens in clinical trials seldomly include pharmacological (kinase) inhibitor drugs, as summarised by Marshall in 2014 (202). This particular treatment strategy in sepsis is still in its infancy, and therapeutic targeting of kinases and phosphatases holds promise towards the development of successful treatment regimens for sepsis patients. This is spurred by technological advancements to analyse kinase and phosphatase activity, that now allow us to determine kinome and phosphatome profiles in cells and tissue during a particular condition (183, 203). This strategy reveals an unbiased selection of kinases and phosphatases with increased or decreased activity, and thereby will help identify potential targets for therapeutic intervention, as well as detecting potential side effects in non-diseased cells and tissues (204).

Many kinase inhibitors interact with the catalytic domain of a kinase, thereby rendering it unable to phosphorylate its substrate. Due to structural homology between kinases, kinase inhibitors tend to not only inhibit the activity of one kinase, but also alter the activity of other types of kinases (205). These so-called off-target effects are commonly seen as an impediment to a broader application of kinase inhibitor treatments, because it can lead to unwanted and unpredictable side-effects (206, 207). Moreover, in some instances the exact target(s) of kinase inhibitor drugs are poorly described. This is exemplified by a recent study revealing that kinase inhibitor OTS964 exerts its function not through its putative target PDZ-binding kinase, but rather *via* cyclin-dependent kinase 11 (208). The misidentification of the target of OTS964 likely stems from RNA interference promiscuity, an obstacle that is circumvented with recently developed CRISPR-based gene knockdown (208). In the development of new generation kinase inhibitor drugs, increasing emphasis is put on enhanced target specificity, while reducing off-target effects (209, 210). Although the reasoning behind this development is valid, it is likely not the way forward for the application of kinase inhibitor drugs for sepsis treatment. Keeping the considerable levels of redundancy between signalling pathways in mind, targeting a single kinase with a highly specific kinase inhibitor may potentially be ineffective, as related pathways will strive to compensate for the loss of activity of that particular kinase (211). Instead, kinase inhibitors with low(er) target specificity can target a wider group of kinases and might therefore be more effective in shutting down a combination of pathways without evoking compensation. This topic is reviewed in more detail elsewhere (209). If specific inhibition of a single kinase is preferred, it is convenient to also have drugs with higher levels of specificity at one’s disposal to satisfy both needs. The field of oncology has a long history with the development of and treatment with kinase inhibitors, which has led to an expanding number of kinase inhibitor drugs that are FDA/EMA-approved (212, 213). Whenever one of these drugs is found to improve the condition of sepsis patients, such repurposed drugs can complete mandatory intermediary steps more rapidly than newly developed drugs before allowing administration of this drug to sepsis patients (214). An example of a repurposed kinase inhibitor drug for sepsis is imatinib. In mouse CLP-sepsis this drug, developed as multi-target kinase inhibitor for anticancer treatment (215), attenuated renal microvascular leakage (216). Both repurposing as well as off-label use of kinase inhibitor drugs are becoming increasingly commonplace in oncology, which could serve as an example for drug development for sepsis (217, 218).

Another important consideration involves the position of the target kinase of choice in its respective signalling cascade. On the one hand, it could be effective to target a kinase that functions during the initiation of the sepsis-induced intracellular signalling cascade, *e.g.* tyrosine kinase receptors, which then inhibits the very start of the signal transduction pathway. On the other hand, a strategy relying on kinases that are further downstream in the signalling cascade has the benefit of only targeting components of the pathway that are deemed injurious. In this way, non-damaging or potential beneficial signalling pathways of the original stimulus remain unaffected, whilst only the harmful pathway is inhibited. Opting for targeting kinases at either the start or end of the signalling cascade will most likely depend on the existence of pathway redundancies, the target cell of interest, the stage of the target disease, and insights in the time frame in which a particular treatment can effectively block signalling in its target cells. During early sepsis, pro-inflammatory mediators will already have had the opportunity to initiate intracellular signalling in various cell types involved in sepsis pathophysiology. Inhibition of kinases after they have already initiated signalling is likely ineffective. Instead, a more effective approach could involve targeting kinases that become activated at later stages of sepsis, considering that it typically requires hours to days to develop clinical symptoms before sepsis can be diagnosed. This also highlights the need for reliable blood biomarkers to detect early sepsis, and to establish the stage of disease progression in patients. More research is required to identify windows of time within which therapeutic treatment with a particular drug of interest would have favourable effects for sepsis patients.

With regard to the administration of kinase inhibitor drugs to patients, another issue that needs to be addressed is the different cell types that are targeted by the treatment. For example, a certain kinase inhibitor treatment might prevent sepsis-induced dysfunction of EC in glomeruli of the kidney upon systemic administration. The drug will also have access to and thus affect cells other than glomerular endothelium. This can lead to altered kinase activity in other microvascular beds in the kidney and in other organs, as well as in non-endothelial cell types throughout the body. Even though some kinases and phosphatases are known to be restrictedly expressed by EC, the majority of kinases and phosphatases is expressed in a multitude of cell types. For that reason, systemic inhibition of kinases or phosphatases could lead to side-effects of the treatment that in a worst-case scenario completely counteract its beneficial effects. Deleterious side-effects of drugs may depend on the activation status of the targeted kinase in these non-target cell and tissue types, but this knowledge is currently lacking. Recent advances in kinome and phosphatome activity arrays (204), as well as phosphoproteomics (219), represent novel opportunities to contribute to our understanding of kinase activation status in different cell and tissue types, and may help predict side-effects of particular drug treatments.

### Improving Translational Research in Sepsis for Patient Benefit

Most research that strives to identify novel druggable targets in sepsis and sepsis-MOF follows the traditional pipeline of *in vitro* studies, followed by animal studies, and in some cases the study next advances to the stage of clinical trials (220). Even though this strategy has led to success in other fields (213), and has also contributed to our current understanding of sepsis, it has not yet led to the development of an effective therapeutic strategy to halt the development of multiple organ failure as a result of sepsis (221). One reason for this might lie in the complexity of sepsis, or that *in vitro* studies are generally a poor predictor of how cell types respond to a certain stimulus or drug *in vivo*. This is particularly true for EC, which exhibit unique gene expression profiles and behaviour when being in their microvascular environment, a feature that is lost when they are isolated and cultured (222). Immortalised endothelial cell lines are the farthest removed from mimicking the *in vivo* situation. This issue is somewhat salvaged by culturing primary EC (223), but also these cells quickly drift into a generic EC phenotype, leading to the loss of their unique *in vivo* characteristics. This loss of unique characteristics is likely a combination of the absence of flow in most *in vitro* settings, lack of interaction with supporting cells such as pericytes and smooth muscle cells, and lack of interaction with bloodborne cells (222).

To maximise the translational value of the available models, we propose that we depart from the linearity and unidirectionality of the traditional research pipeline and adopt an approach that facilitates going back and forth between *in vitro*, *in vivo*, *ex vivo*, and between animal models and patients. By no means does this suggest omitting any of the aforementioned steps, but it rather ensures that information on the involvement of a target molecule in the disease process is maximised to enable informed decision-making. These intermediary steps include organ-on-a-chip, organoids, *ex vivo* precision cut organ slices, and patient biopsies. In short, organ-on-a-chip are microdevices containing different cell types that can mimic key characteristics of particular organs *in vitro* (224). Organoids rely on self-organisation of the cells to form more complex 3D structures compared to organ-on-a-chip, which are also used to study particular aspects of organ behaviour *in vitro* (225). To identify the involvement of a target kinase in the development of sepsis in EC, organ-on-a-chip and organoids could be implemented to study the activity of the target kinase under homeostatic and pro-inflammatory conditions. This would increase our understanding of the various cell types within an organ that express the kinase of interest. Innovations to enable (vascular) flow in these models are under development (226), and vascular flow in kidney organoids has been described as a prerequisite for proper vascularisation to take place (227). Further developments are required for these models to become relevant tools to study endothelial heterogeneity, as the kidney cortex alone consists of distinct microvascular beds, *i.e.* arterioles, glomeruli, peritubular capillaries, and post-capillary venules (6), which represent a level of structural complexity not yet present in kidney organoids. Post-mortem organ biopsies of both sepsis and non-sepsis donors could help determine the presence of the target kinase in different organs using protein detection tools ranging from ELISA to proteomics. In addition, using kinase activity arrays (204) and phosphoproteomics (219) to compare kinase activation status in biopsies from both sepsis and non-sepsis donors could hint towards the involvement of the target kinase in sepsis.

Precision cut tissue slices (PCTS) represent small sections of viable human tissue, in which cell types can be studied while situated in their original pathophysiological niche (228, 229). Importantly, the retained endothelial heterogeneity of the microvascular compartments in these organs provides valuable information on microvascular compartment-specific responses to certain stimuli that would be unattainable *in vitro* (230). One could also imagine a setting in which PCTS from both humans and experimental animals are compared for their response to a particular kinase inhibitor drug. If the response of the human PCTS differs greatly from that of animal PCTS, continuation of this study in that particular animal model might not be worthwhile, as this indicates that the effects of drug treatment in animal will not be representative of effects in humans. In the case of biomarker development in conjunction with novel therapies, a biomarker of sepsis found in a human PCTS model can be validated in human patient blood or urine. If its presence is confirmed, it can be determined whether this is also the case in the animal model utilised for sepsis. If so, one can determine expression levels of the biomarker in *e.g.* mice upon treatment with a novel drug, and next translate this back to the human PCTS model. In this manner, there is constant crosstalk between models, and incongruencies caused by differences in models or differences between species are discovered at an early stage. These tools might offer earlier confirmation that a target and/or biomarker of interest truly is involved in the development of sepsis or indicate that pursuit of the target is not worthwhile. In the end, all experimental models come with their own advantages and disadvantages, and this realisation can help select combinations of models that will answer the research question at hand. The traditional research pipeline has not resulted in a drug to effectively treat sepsis, which can be viewed as a confirmation that *in vitro* and animal studies alone are poor predictors of pathophysiology in sepsis patients. Inclusion of organ-on-a-chip, organoids, precision cut organ slices, and patient biopsies when designing novel drug therapies will enable making better-informed decisions and will allow resources to be spent on more promising targets and biomarkers.

There are additional ways in which sepsis animal studies and clinical trials can be further optimised. For instance, many animal studies and clinical trials with sepsis patients tend to focus on a limited number of readouts, including IL-6 plasma levels as a parameter of inflammation, or biomarkers indicating dysfunction of a single organ. Sepsis is by definition a systemic disease, that concurrently induces dysfunction of multiple organs. Using an animal sepsis model to study the effects of an experimental drug only on *e.g.* sepsis-induced acute lung injury is an interesting quest in its own right but fails to acknowledge the fact that the drug might also influence the disease trajectory in organs that are not of primary interest. The drug might be reported as a promising perspective for sepsis patients because it reduced acute lung injury, while at the same time effects on *e.g.* kidney and liver were not reported, or vice versa. To gain a more complete overview of the effects that drugs have on sepsis-related pathophysiology, effects of drugs should be assessed in brain, heart, lung, liver, and kidney, as they are most commonly affected in sepsis. Such an approach would vastly increase the quantity and quality of information that is generated per animal. This is especially true for vascular research, because the extensive heterogeneity of EC between and within organs may underlie microvascular subsets responding divergently from one another (6). By studying and reporting the effects of drugs on all major organs, studies will become increasingly informative, while simultaneously enhancing the translatability of this translational research.

This ties in with the previously discussed PROWESS-SHOCK clinical trial, which eventually showed not to improve survival of sepsis patients despite promising preceding findings. Firstly, sepsis treatment should not negatively affect non-diseased tissue in such a way that it worsens the overall condition of the patient or studied animal. In the case of PROWESS-SHOCK, patients receiving treatment initially showed attenuated systemic inflammation and improved survival (197), positive effects that were not found in a later study (198). The reported lack of improved survival in the latter study cannot be fully explained, but complications caused by the effects of the drug on coagulation and subsequent bleeding complications may have played a role. Gaining an indication of whether the positive effects of a drug exceed the negative side-effects before entering clinical trials is exactly what the sepsis and sepsis-MOF research field is struggling with. The variation between and within different sepsis animal models (221, 231) only further adds to the already limited translatability of preclinical research to the clinical phase (220). In addition, coagulopathy exemplifies the differences in symptoms between human sepsis and animal models of sepsis, as it is a common condition in sepsis patients, yet many animal models of sepsis fail to produce symptoms mimicking sepsis-associated coagulopathy (232), thereby considerably reducing their translational value. Secondly, the PROWESS-SHOCK study forces us once more to acknowledge the extreme heterogeneity of sepsis patients, which is underscored by the recent description of the existence of four clinical sepsis phenotypes. These phenotypes are characterised based on a variety of host-response biomarkers, including markers of abnormal coagulation (200). Coagulopathy affects individual patients to different extents at different times (14). Therefore, a drug with coagulation-modulatory side-effects might be harmless or even beneficial for one patient, while being detrimental to a patient with a different clinical sepsis phenotype.

There are two final points that may improve the translatability of animal studies or clinical trials for sepsis. First of all, a shift from drug pre-treatment to drug intervention studies in animal models of polymicrobial sepsis will help assess drug effectiveness in a setting that is clinically more relevant. While pre-treatment with a drug of choice before inducing sepsis can indicate the involvement of the target under study in the onset and progression of sepsis, it does not provide any information on applicability of the drug for sepsis treatment. Hence, when performing animal studies with novel drugs aimed to ameliorate sepsis, intervention studies need to take up a considerable portion of the research, as these are better representatives of the performance of the drug in the complexity of the pathophysiological process. This is tying in with the second point, that deals with the importance of providing proof of the activity of the drug in its target tissue and its targeted cells. When a kinase inhibitor drug is administered in an animal sepsis model to ameliorate acute lung injury by targeting a kinase in lung EC, it is insufficient to simply report the extent of acute lung injury or show altered levels of soluble markers of endothelial dysfunction in plasma. Neither parameter provides the necessary proof that the pulmonary vasculature was indeed effectively targeted by the drug. Instead, one might consider quantification of inflammatory proteins expressed by target cells in the target tissue, in addition to quantifying activity levels of the target kinase of the inhibitor in EC. In addition, tools such as laser microdissection can be used to zoom in on individual microvascular compartments, for instance to study their unique gene expression patterns in both health and disease (22). Laser microdissected material is also compatible with (mi)RNA sequencing platforms, allowing for high-throughput characterisation of microvascular compartment-specific signatures (233). These types of analyses will create better insights into the molecular mechanisms of drugs and will enable identification of subsets of cells that are, or are not, affected by the drug treatment. Anything other than direct evidence is suboptimal, because ambiguity about the pharmacological mechanism of a drug at this phase is bound to lead to unforeseen side-effects of the drug at subsequent stages of development.

## Conclusion

Despite considerable advances in our understanding of sepsis pathophysiology, clinicians and researchers have not yet been able to develop effective drug treatment regimens to prevent or treat sepsis and sepsis-MOF. In this review, we set out to highlight the role of endothelial cells in sepsis, as these represent an understudied cellular player in the pathophysiology of sepsis, and we provided a comprehensive overview of the major signal transduction pathways involved in sepsis-induced endothelial cell activation and dysfunction. We discussed animal studies and clinical trials intervening in these pathways, with emphasis on how the involvement of interconnected kinases and phosphatases form sepsis-associated intracellular signalling networks. Despite the potential that kinase (and phosphatase) inhibitor drugs hold as a treatment for sepsis, several challenges need to be overcome before their applicability for sepsis patients can be assessed. The molecular mechanisms involved in sepsis are so complex, that it seems unlikely that any approach targeting a single cell type or a single pathway will succeed. Yet through adopting a research design with which the amount of information obtained from a single study increases, while simultaneously enhancing the translatability of outcomes in the lab to clinical applications *via* intermediary models including organoids and precision cut tissue slices, the overarching goal of uncovering effective therapeutic treatments for sepsis and sepsis-MOF becomes within reach.

## Author Contributions

All authors listed have made a substantial, direct, and intellectual contribution to the work and approved it for publication.

## Funding

This project is co-financed by the Ministry of Economic Affairs and Climate Policy by means of the PPP-allowance made available by the Top Sector Life Sciences & Health to stimulate public-private partnerships (#6334, MM).

## Conflict of Interest

GM is cofounder and CSO/CT of Vivomicx, which provides laser microdissection analysis services. Vivomicx financially contributes to the PPP-allowance.

The remaining authors declare that the research was conducted in the absence of any commercial or financial relationships that could be construed as a potential conflict of interest.

## Publisher’s Note

All claims expressed in this article are solely those of the authors and do not necessarily represent those of their affiliated organizations, or those of the publisher, the editors and the reviewers. Any product that may be evaluated in this article, or claim that may be made by its manufacturer, is not guaranteed or endorsed by the publisher.

## Footnotes

1 This review is not a systematic review. Instead, it cites articles found in online databases using various search terms that we found relevant to provide insight into which intracellular signalling pathways have been described to be activated in endothelial cells when exposed to sepsis-associated stimuli that may serve as targets for therapeutic intervention.

## Glossary

ABIN-2: A20-binding inhibitor of NF-κB activation-2
AKT: protein kinase B
Angpt1: angiopoietin-1
Angpt2: angiopoietin-2
APC: activated protein C
AP-1: activator protein 1
Axl: Axl receptor tyrosine kinase
CASP: colon ascendens stent peritonitis
CLP: cecal ligation and puncture
c-Fos: cellular Fos proto-oncogene
c-Jun: cellular Jun proto-oncogene
EC: endothelial cell
EPCR: endothelial protein C receptor
ERK: extracellular signal-regulated kinase
FAK1: focal adhesion kinase 1
Gas6: growth arrest specific 6
HDL: high-density lipoprotein
ICAM-1: intercellular adhesion molecule 1
IKK: IκB kinase
IL-1β: interleukin 1 beta
IL-6: interleukin 6
IκB: inhibitor of κB
JNK: c-Jun N-terminal kinase
LPS: lipopolysaccharide
MCP-1: monocyte chemoattractant protein 1
mDia: mammalian Diaphanous-related formin Dia
miR: microRNA
MLC: myosin light chain
MLCK: myosin light chain kinase
MLCP: myosin light chain phosphatase
MMP14: matrix metalloproteinase-14
NF-κB: nuclear factor kappa-light-chain-enhancer of activated B cells
p38: p38 mitogen-activated protein kinase
PAK: p21-activated kinase
PAR1: protease-activated receptor 1
PCI: peritoneal contamination and infection
PI3K: phosphoinositide 3-kinase
Rac1: Ras-related C3 botulinum toxin substrate 1
Ras: Ras protein subfamily
RhoA: Ras homolog gene family, member A
RIG-I: retinoic acid-inducible gene I
ROCK: Rho-associated protein kinase
S1P: sphingosine-1-phosphate
S1PR1: sphingosine-1-phosphate receptor 1
S1PR2: sphingosine-1-phosphate receptor 2
sepsis-MOF: sepsis-associated multiple organ failure
SK1: sphingosine kinase 1
Src: Src protein tyrosine kinase
Tie1: tunica intima endothelial kinase 1
Tie2: tunica intima endothelial kinase 2
TLR4: Toll-like receptor 4
TM: thrombomodulin
TNFR1: tumour necrosis factor alpha receptor 1
TNFR2: tumour necrosis factor alpha receptor 2
TNFα: tumour necrosis factor alpha
VCAM-1: vascular endothelial adhesion molecule 1
VEGF: vascular endothelial growth factor
VEGFR2: vascular endothelial growth factor receptor 2
VE-cadherin: vascular endothelial cadherin
VE-PTP: vascular endothelial protein tyrosine phosphatase
vWF: von Willebrand Factor
