# Supplementary material for: Unlocking the Untapped Potential of Endothelial Kinase and Phosphatase Involvement in Sepsis for Drug Treatment Design
Source: Front Immunol. 2022 May 13;13:867625. doi: 10.3389/fimmu.2022.867625 (PMC9136877; doi:10.3389/fimmu.2022.867625)
Supplement: Supplementary file 1 [file Table_1.pdf]

Supplementary Table 1. Summary of consulted references to construct the signal transduction pathway schematics

NF-κB-related connections						
**Interaction**	**In vitro/vivo**	**Endogenous pathway or overexpression**	**Treatment / Intervention**	**Effect**	**Miscellaneous**	**Author**
TNFα activates IKK	In vitro (HUVEC)	Endogenous pathway	TNFα	TNFα increased IKK protein phosphorylation status	30 min TNFα stimulation	Zheng et al. (2013)
LPS activates IKK	In vitro (HPMEC)	Endogenous pathway	LPS	LPS increased IKKβ protein phosphorylation status	12 min LPS stimulation	Menden et al. (2013)
LPS activates RIG-I + TLR4	In vitro (HUVEC)	Endogenous pathway	LPS + RIG-I siRNA + TLR4 siRNA	LPS-induced increases in mRNA levels of E-selectin/VCAM-1/ICAM-1/IL-6/IL-8 were reduced similarly by either RIG-I siRNA or TLR4 siRNA	4 h LPS stimulation	Moser et al. (2016)
RIG-I activates NF-κB pathway	In vitro (HUVEC)	Endogenous pathway	LPS + RIG-I siRNA	LPS-induced reduction in IκB protein levels was abolished by RIG-I siRNA	30 min LPS stimulation	
TNFα activates RIG-I	In vitro (HUVEC)	Endogenous pathway	TNFα + RIG-I siRNA	TNFα-induced increase in mRNA levels of E-selectin/VCAM-1/ICAM-1/IL-6/IL-8 was decreased by RIG-I siRNA	4 h TNFα stimulation	
TNFα activates RhoA	In vitro (Bend.3)	Transduced with dominant negative RhoA	TNFα	TNFα increased WT RhoA activation levels + TNFα-induced TEER reduction was alleviated in cells expressing dominant negative RhoA	Effect of TNFα on RhoA activation visible after 1 min, effect on TEER between 0.5-3 h	Peng et al. (2011)
Thrombin-activated RhoA/ROCK activates IKK	In vitro (HUVEC)	Endogenous pathway	Thrombin + pharmacological ROCK-inhibitor Y27632	Thrombin increased IKK protein phosphorylation status, which was reverted by Y27632 pretreatment	1 h thrombin stimulation	Anwar et al. (2004)

Rac1/RhoA GTPases-related connections						
**Interaction**	**In vitro/vivo**	**Endogenous pathway or overexpression**	**Treatment / Intervention**	**Effect**	**Miscellaneous**	**Author**
Rac1 inhibits RhoA	In vitro (HMVEC-Lung)	Endogenous pathway	Angpt1 + p190 RhoGAP siRNA	Angpt1 activated Rac1 and inactivated RhoA, but RhoA activity increased with p190 RhoGAP siRNA pretreatment	30 min Angpt1 stimulation	Mammoto et al. (2007)
	In vivo (mice)	Endogenous pathway	Angpt1 + LPS + p190 RhoGAP siRNA	Angpt1 protected against LPS-induced vascular leakage in the lung, but this effect was blocked by p190 RhoGAP siRNA pretreatment	8 h after first Angpt1 dose, mice were co-injected with Angpt1 and LPS. Mice were sacrificed 16 h after second injection	
RhoA inhibits Rac1 via ROCK	In vitro (HEK293)	Transfected with constitutively active ROCK ($ROCK^{\Delta3}$) and FilGAP or FilGAP with mutated phosphorylation sites ($FilGAP^{STA}$)		Activated Rac1 levels were decreased by $ROCK^{\Delta3}$, this decrease was even more pronounced in presence of FilGAP. This additional decrease in Rac1 activity was abolished in the presence of $FilGAP^{STA}$		Ohta et al. (2006)
RhoA activates ROCK	In vitro (HUVEC)	Endogenous pathway	Thrombin + Rho-binding domain of ROCK, or catalytic domain of ROCK	Thrombin-induced formation of stress fibres was inhibited by Rho-binding domain of ROCK. Administration of catalytic domain of ROCK mimicked effects of thrombin.	15 min thrombin stimulation	Essler et al. (1998)
ROCK phosphorylates MLCP	Proteins isolated from human platelets	Constitutively activated ROCK (GST-ROCK)	Staurosporine (pharmacological inhibitor of ROCK)	Staurosporine reverted GST-ROCK-induced increase in phosphorylation of MLCP-subunit and reduction in MLCP activity	20 min GST-ROCK stimulation	Nakai et al. (1997)
Rac1 inhibits MLCK	In vitro (HeLa)	Transfected with constitutively active PAK1 (T423E)		T423E decreases MLCK activity, indicated by decreased MLC protein phosphorylation status	PAK1 is downstream of Rac1	Sanders et al. (1999)

Cortical actin assembly protects barrier function	In vitro (HUVEC)	Endogenous pathway	Cytochalasin D (depolymerises actin) + FSK (pharmacological adenylyl cyclase activator)	FSK enhances endothelial barrier function via increased cortical actin assembly, but fails to do so after pre-treatment with cytochalasin D	30 min FSK treatment	Fukuhara et al. (2005)
Rac1 leads to cortical actin assembly	In vitro (porcine pulmonary artery endothelial cells; PPAEC)	Transfected with constitutively activated Rac1 mutant (V12Rac1)	Hypoxia	V12Rac1 prevented hypoxia-induced stress fibre formation and decreased permeability	After 48 h (normoxia), V12Rac1 increased permeability as a result of a loss of cortical actin	Wojciak-Stothard et al. (2005)
RhoA-induced stress fibre formation disrupts barrier function	In vitro (PPAEC)	Transfected with dominant negative RhoA mutant (N19RhoA)	Hypoxia	Hypoxia-induced stress fibre formation and permeability were abolished in cells expressing N19RhoA		

AP-1-related connections						
**Interaction**	**In vitro/vivo**	**Endogenous pathway or overexpression**	**Treatment / Intervention**	**Effect**	**Miscellaneous**	**Author**
TNFα activates JNK	In vitro (HUVEC)	Endogenous pathway	TNFα	TNFα increased JNK activity, illustrated by increased c-Jun protein phosphorylation status	0-60 min TNFα stimulation	Surapisitchat et al. (2001)
TNFα activates p38				TNFα increased p38 activity, illustrated by increased p38 protein phosphorylation status		
TNFR1 activates Ras/ERK pathway	In vitro (HMVEC-dermal)	Endogenous pathway	48 h recovery after Raf antisense oligonucleotide treatment, followed by TNFα	TNFα-induced increase in ELK protein phosphorylation status was reduced by Raf antisense oligonucleotides pretreatment	15 min TNFα stimulation. ELK and RAF are downstream of ERK and Ras, respectively	Xu et al. (1998)
TLR4 activates ERK	In vitro (HUVEC)	Endogenous pathway	LPS	LPS increased ERK activity	2 h LPS stimulation	Jersmann et al. (2001)
TLR4 activates JNK				LPS increased JNK activity		
TLR4 activates p38				LPS increased p38 activity		
RhoA activates p38	In vitro (HUVEC)	Endogenous pathway	LPS + RhoA siRNA	LPS-induced increase in p38 protein phosphorylation status was reduced after RhoA knockdown	0-60 min LPS stimulation	Guo et al. (2012)
	In vitro (HEK293)	Transfection with m1 muscarinic acetylcholine receptor, GST-MKK6, and dominant negative RhoA mutant RhoT19N	Carbachol	Carbochol-induced increase in p38 protein phosphorylation status was abolished by RhoT19N		Yamauchi et al. (2001)
Rac1 activates p38		Transfection with m1 muscarinic acetylcholine receptor, GST-MKK6, and dominant negative		Carbochol-induced increase in p38 protein phosphorylation status was abolished by RacT17N		

		Rac1 mutant RacT17N				
RhoA activates JNK	In vitro (HEK293)	Transfection with GST-MKK4, Gβ1, Gγ2, and dominant negative RhoA mutant RhoT19N		Gβ1/ Gγ2-induced increase in c-Jun protein phosphorylation status and increased MKK4 activity were abolished by RhoT19N		Yamauchi et al. (1999)
Rac1 activates JNK		Transfection with GST-MKK7, Gβ1, Gγ2, and dominant negative Rac1 mutant RacT17N		Gβ1/ Gγ2-induced increase in c-Jun protein phosphorylation status and increased MKK7 activity were abolished by RacT17N		
ERK induces c-Fos transcription	In vitro (HeLa)	Transfection with activated Raf (RafBxB), ERK2, c-Fos reporter pWTGL3, and dominant negative CREBm1		RafBxB/ERK2-induced increase in c-Fos transcription was inhibited by CREBm1	CREB is located downstream of ERK and is required for transcriptional activation of c-Fos	Wang et al. (2000)
ERK phosphorylates c-Fos	In vitro (non-specified; presumably NIH 3T3)	Overexpressing WT c-Fos or c-Fos-m	MEK EE (constitutively active MEK1) + ERK2 + GST-Pin1	MEK EE + ERK2 induced c-Fos protein phosphorylation and subsequent interaction with Pin1 in WT c-Fos, but not in c-Fos-m	c-Fos-m had mutated ERK-phosphorylation sites	Monje et al. (2005)
JNK induces c-Jun transcription	In vitro (MEF)	Cells derived from $jnk^{-/-}$ knockout mice	EGF	EGF-stimulated increase in c-Jun mRNA and protein levels in WT MEFs was inhibited in $jnk^{-/-}$ cells	0-120 min EGF stimulation	Kayahara et al. (2005)
p38 inhibits JNK-induced c-Jun transcription			EGF + pharmacological p38 inhibitor SB203580	EGF-stimulated increase in c-Jun mRNA and protein levels in WT MEFs was further increased by SB203580 pretreatment. No effect of SB203580 was observed in $jnk^{-/-}$ cells. SB203580 increased JNK activity.		
ERK induces c-Jun transcription			EGF + pharmacological	EGF-stimulated increase in c-Jun mRNA and protein levels in		

			ERK inhibitor PD184352	WT MEFs was inhibited by PD184352 pretreatment for early (15-30m) but not for late (60m) c-Jun mRNA levels		
JNK phosphorylates c-Jun	In vitro (MEF)	Cells derived from jnk$^{-/-}$ knockout mice	TPA	TPA induced c-Jun protein phosphorylation at several sites was inhibited in jnk$^{-/-}$ cells	30 min TPA stimulation	Morton et al. (2003)
p38 inhibits ERK	In vitro (HUVEC)	Endogenous pathway	Angpt1 + pharmacological p38 inhibitor SB203580	SB203580 pretreatment further increased Angpt1-induced increase in ERK protein phosphorylation status	5-60 min Angpt1 stimulation	Abdel-Malak et al. (2008)
p38 induces c-Jun transcription	In vitro (HUVEC)	Endogenous pathway	Pharmacological PI3K inhibitor LY294002 + pharmacological p38 inhibitor SB203580	LY294002 increased ATF-2 protein phosphorylation status, which was abolished upon treatment with SB203580	5 h inhibitor treatment. ATF-2 protein is involved in c-Jun transcription	Gratton et al. (2001)
p38 induces c-Fos transcription	In vitro (COS-7)	Isolated purified p38 from UV-stimulated COS-7	Performed kinase assay with Elk1	Activated p38 from UV-treated COS-7 cells induced phosphorylation of Elk1	Elk1 protein is involved in c-Fos transcription	Whitmarsh et al. (1997)
p38 phosphorylates c-Jun	In vitro (HUVEC)	Endogenous pathway	PMA + pharmacological p38 inhibitor SB202190	PMA-induced c-Jun phosphorylation at S63/S73 was abolished after pretreatment with SB202190	10 min PMA stimulation	Humar et al. (2007)
p38 phosphorylates c-Fos	In vitro (HEK293)	Transfection with pCEFL-HA-SAPKs (p38α/β/γ/δ) and pCEFL-GFP-c-Fos TAD with or without MKK6		p38(α/β/γ/δ) shifted c-Fos TAD detected by anti-GFP antibodies for all p38 isotypes in presence of MKK6, indicating c-Fos protein phosphorylation	MKK6 is an upstream activator of p38	Tanos et al. 2005
JNK induces c-Fos transcription	In vitro (HeLa)	Transiently transfected with MEKK1		MEKK1 increased JNK protein phosphorylation status, which corresponded with c-Fos mRNA levels	MEKK1 is an upstream activator of JNK	Cavigelli et al. (1995)
TNFα activates AKT and PI3K	In vitro (HUVEC)	Endogenous pathway	TNFα	TNFα increased AKT and PI3K protein phosphorylation status	0-240 min TNFα stimulation	Lo et al. (2014)
LPS activates AKT and PI3K	In vitro (HUVEC)	Endogenous pathway	LPS	LPS increased AKT and PI3K protein phosphorylation status	0-60 min LPS stimulation	Xu et al. (2013)

APC/S1P-related connections						
**Interaction**	**In vitro/vivo**	**Endogenous pathway or overexpression**	**Treatment / Intervention**	**Effect**	**Miscellaneous**	**Author**
APC inhibits thrombin	Human plasma	Endogenous pathway	APC	APC decreased thrombin protein levels in plasma		Nicolaes et al. (1997)
APC activates PAR1	In vitro (HUVEC)	Endogenous pathway	APC	APC induced gene expression profile comparable to that induced by PAR1 agonist		Riewald et al. (2002)
APC-PAR1 activates SK1	In vitro (HUVEC)	Endogenous pathway	APC + SK1 siRNA or S1PR1 siRNA	Barrier protective effect of APC-PAR1 was lost upon knockdown of SK1 or S1PR1	3 h APC stimulation	Feistritzer et al. (2005)
Thrombin-PAR1 activates RhoA	In vitro (HUVEC)	Endogenous pathway	PAR1-activating peptides (PAR1-AP)	Activated RhoA protein levels were increased after PAR1-AP treatment	20 min PAR1-AP stimulation	Vouret-Craviari et al. (2003)
NF-κB transcription inhibits TM	In vitro (BAEC)	Endogenous pathway	TNFα	TNFα lowered TM protein levels and activity	6-8 h TNFα stimulation	Moore et al. (1989)
	In vitro (HUVEC)	Endogenous pathway	TNFα + pharmacological NF-κB blockade (parthenolide)	TNFα-mediated TM downregulation was inhibited at mRNA and protein level after parthenolide treatment	16 h TNFα stimulation	Sohn et al. (2005)
	In vivo (mice)	Plasmids with IκB super-repressor (IκBsr)	TNFα + pre-treatment with IκBsr plasmids for NF-κB blockade	TNFα-mediated TM downregulation was inhibited at mRNA and protein level in IκBsr-transduced animals	TM levels in whole lung extracts 16 h after TNFα injection via intratracheal installation	
	In vitro (BAEC)	Endogenous pathway	TNFα + pharmacological NF-κB blockade (parthenolide)	TNFα-mediated TM downregulation was inhibited at protein level	10 h TNFα stimulation	Lin et al. (2010)
TM/EPCR/thrombin complex activates PC	In vitro (HUVEC)	Endogenous pathway	EPCR-inhibitory antibodies	EPCR-inhibitory antibody decreased APC protein levels		Stearns-Kurosawa et al. (1996)
	In vivo (baboons)	Endogenous pathway	Thrombin + EPCR-inhibitory antibodies	EPCR-inhibitory antibody decreased APC protein levels in plasma	APC levels were monitored 2 h	Taylor et al. (2001)

NF-κB activates SPP2	In vitro (HUVEC)	Endogenous pathway	TNFα + RelA siRNA	TNFα-mediated SPP2 upregulation was inhibited at mRNA level by RelA knockdown	8 h TNFα stimulation	Mechtcheriakova et al. (2007)
SPNS2 transports S1P	In vivo (mice)	Endogenous pathway	Genetic disruption of SPNS2 gene	SPNS2-deficient mice showed reduced S1P plasma levels		Hisano et al. (2012)
	In vitro (HUVEC/HPAEC)	Endogenous pathway	SPNS2 siRNA	Loss of SPNS2 reduced S1P plasma levels		
S1P associates with HDL/albumin	Human blood	Endogenous pathway	S1P	S1P predominantly bound to HDL and albumin		Aoki et al. (2005)
HDL-bound S1P activates S1PR1	In vitro (HUVEC)	Endogenous pathway	apoM$^+$ HDL containing S1P + pharmacological S1PR1 inhibitor VPC44116	apoM$^+$ HDL activated downstream targets of S1PR1, which was reversed in the presence of VPC44116	10 min apoM$^+$ HDL stimulation	Christoffersen et al. (2011)
	In vivo (mice)	Genetic overexpression of apoM$^+$ or apoM$^{-/-}$ HDL		apoM$^{-/-}$ HDL mice exhibited decreased endothelial lung barrier function compared to apoM$^+$ HDL mice	Endothelial lung barrier function was assessed via Evans Blue staining	
AKT phosphorylates S1PR1-T236	In vitro (HUVEC)	Endogenous pathway		AKT associated with S1PR1 and phosphorylated T236		Lee et al. (2001)
S1PR1-T236 phosphorylation activates Rac1	In vitro (CHO)	Transduced with S1PR1-T236 mutant	S1P	S1P/S1PR1-induced Rac1 activation was inhibited by S1PR1-T236 mutant		
S1PR1 activates ERK	In vitro (HUVEC)	Endogenous pathway	FTY720P (S1PR agonist)	ERK protein phosphorylation status was increased by FTY720P treatment	5 h FTY720P stimulation	Mullershausen et al. (2009)
S1PR2 activates RhoA	In vitro (HUVEC)	Endogenous pathway	S1P (10 μM) + pharmacological S1PR1 inhibitor VPC23019 / S1PR2 inhibitor JTE013	S1P increased RhoA activity, which was reverted by JTE013, but not by VPC23019	S1P increased RhoA activity after 2-5 min, and returned to control levels after 10 min	Li et al. 2015
S1P stimulates endothelial barrier function via Rac1	In vitro (HUVEC)	Endogenous pathway	S1P (0.5 μM) + pharmacological Rac1 inhibitor NSC23766	S1P-mediated increase in TEER was hampered by NSC23766	Effect of S1P on TEER was visible after 10 min	

S1P inhibits endothelial barrier function via RhoA	In vitro (HUVEC)	Endogenous pathway	S1P (10 µM) + pharmacological ROCK inhibitor H1152	S1P-mediated decrease in TEER was attenuated by H1152		
SPP2 dephosphorylates S1P	In vitro (HEK293)	Overexpression of SPP2	S1P	SPP2 overexpressing cells displayed lower levels of S1P and higher levels of Sph		Ogawa et al. (2003)
SPL degrades S1P	In vivo (mouse)	Endogenous pathway	Pharmacological SPL inhibitor FTY720	FTY720 induced an increase in S1P levels in spleen and thymus		Bandhuvula et al. (2005)
LPP dephosphorylates S1P	In vitro (ras-transformed rat2 fibroblasts)	Overexpression of LPP		Increased S1P dephosphorylation during overexpression of LPP		Jasinska et al. (1999)

Angpt/Tie2-related connections						
**Interaction**	**In vitro/vivo**	**Endogenous pathway or overexpression**	**Treatment / Intervention**	**Effect**	**Miscellaneous**	**Author**
Tie1 activates Tie2	In vivo (mice)	Endogenous pathway	LPS	Tie1 is cleaved after LPS injection, leading to reduced Tie2 signaling (AKT protein phosphorylation status)	Sacrificed 16 h after LPS administration. Studied in lung lysate	Korhonen et al. (2016)
Tie2 activates ABIN-2	In vitro (HUVEC)	Transfection with plasmids for FLAG-ABIN-2	Angpt1	Angpt1-dependent activation of Tie2 induced Tie2/ABIN-2 complex formation shown by coimmunoprecipitation	10 min Angpt1 stimulation	Hughes et al. (2003)
ABIN-2 inhibits IKK	In vitro (HEK293T)	Transfection with plasmids for ABIN-2 or ABIN-2 mutant with defect IKK binding domain	TNFα	ABIN-2 prevented TNFα-induced NF-κB signaling, but mutated ABIN-2 did not	4 h TNFα stimulation	Liu et al. (2004)
Tie2 activates Rac1 via PI3K	In vitro (HMVEC-L)	Endogenous pathway	Angpt1 + pharmacological PI3K inhibitor LY294002	Angpt1-induced increase in activated Rac1 protein levels was reverted by LY294002	30 min Angpt1 stimulation	Mammoto et al. (2007)
Tie2 activates mDia via RhoA	In vitro (SVEC)	Endogenous pathway	Angpt1 + RhoA siRNA	Interaction of mDia and Src was abolished in absence of Angpt1 or by RhoA siRNA	15 min Angpt1 stimulation	Gavard et al. (2008)
Tie2 activates ERK via Ras	In vitro (HUVEC)	Endogenous pathway	Angpt1	Angpt1 increased ERK1 and ERK2 protein phosphorylation status	0-60 min Angpt1 stimulation	Kim et al. (2002)
ERK activate SK1	In vitro (HEK293)	Overexpressing SK1	TNFα or PMA + pharmacological ERK1/2 inhibitor U0126	TNFα/PMA-induced increase in ERK protein phosphorylation status and SK1 activity were abolished by U0126 treatment	30 min TNFα/PMA stimulation	Pitson et al. (2003)
APC activates Tie2	In vivo (mice)	Endogenous pathway	APC + LPS + Tie2 inhibitor (Tie2-I)	APC protection against LPS-induced leakage after 4 h and 24 h in lung and kidney was		Minhas et al. (2017)

				reversed by Tie2-inhibitor treatment		
	In vitro (HUVEC)	Endogenous pathway	APC	APC treatment increased Tie2 protein phosphorylation status	2 min APC stimulation	
Angpt1/2 inhibit TM-dependent formation of APC	In vitro (HUVEC)	Endogenous pathway	Protein C + thrombin + Angpt1 or Angpt2	Relative levels of APC were decreased in a concentration-dependent manner in presence of Angpt1 or Angpt2	60 min Angpt1/2 stimulation	Daly et al. (2018)
	Isolated proteins	Endogenous pathway	Soluble TM + thrombin + Angpt1 or Angpt2	Levels of thrombin bound to TM are reduced in presence of Angpt1 or Angpt2		
MMP14 cleaves Tie2	In vitro (HUVEC)	Endogenous pathway	MMP14 siRNA	MMP14 knockdown lowered cleaved soluble Tie2 (sTie2) levels		Thamm et al. (2018)
TNFα activates MMP14	In vitro (HUVEC)	Endogenous pathway	TNFα + MMP14 siRNA	TNFα increased sTie2 levels, which was reverted by MMP14 knockdown	24 h TNFα stimulation	
VE-PTP dephosporylates Tie2	In vitro (HUVEC/HEK293)	Transfected with VE-PTP-GFP and Tie2-FLAG	Pharmacological VE-PTP-inhibitor AKB-9785 + Angpt2	Angpt2 failed to phosphorylate Tie2 in presence of VE-PTP (HEK293), but increased Tie2 protein phosphorylation status after AKB-9785 treatment (HUVEC)		Souma et al. (2018)

VEGF/VEGFR2-related connections						
Interaction	In vitro/vivo	Endogenous pathway or overexpression	Treatment / Intervention	Effect	Miscellaneous	Author
AKT is downstream of Src via PI3K	In vitro (HCAEC)	Endogenous pathway	VEGF + p47 siRNA (p47 is essential for VEGFR2 -> Src signaling)	VEGF-induced increase in Src and AKT protein phosphorylation status were abolished by p47 knockdown	0-15 min VEGF stimulation	Lee et al. (2011)
VEGFR2 activates ERK via Ras				VEGF-induced increase in ERK protein phosphorylation status was unaffected by p47 knockdown		
VEGF activates PI3K/AKT via VEGFR2/Src/Axl	In vitro (porcine aortic endothelial cells stably expressing human VEGFR2; PAE/KDR)	Cells were stably expressing human VEGFR2	VEGF + Axl shRNA	VEGF-induced increase in AKT protein phosphorylation status was abolished in Axl shRNA treated cells compared to empty vector control	0-20 min VEGF stimulation	Ruan et al. (2012)
VEGFR2 activates Vav2 via Src	In vitro (SVEC)	Endogenous pathway	VEGF + pharmacological VEGFR2 inhibitor su1498	VEGF-induced increase in Src protein phosphorylation status was abolished by su1498 treatment	10 min VEGF stimulation	Gavard et al. (2006)
			VEGF + Src siRNA	VEGF-induced increase in Vav2 protein phosphorylation status was abolished by Src knockdown		
Vav2 activates PAK via Rac1			VEGF + Vav2 shRNA	VEGF-induced increase in activated Rac1 protein levels was abolished by Vav2 shRNA treatment	5 min VEGF stimulation	
	In vitro (COS-7)	Transfection with PAK	Isolated Rac1 proteins	Rac1 increased PAK protein phosphorylation status		Knaus et al. (1998)
PAK phosphorylates VE-cadherin	In vitro (SVEC)	Transfection with auto-inhibitory domain of PAK (PID)	VEGF	VEGF-induced increase in VE-cadherin protein phosphorylation status was abolished by PID transfection	5 min VEGF stimulation	Gavard et al. (2006)

VE-cadherin phosphorylation leads to its internalisation	In vitro (SVEC)	Transfection with WT or non-phosphorylatable VE-cadherin (S665V)	VEGF	VEGF-induced increase in WT VE-cadherin protein internalisation was inhibited in S665V expressing cells	30 min VEGF stimulation	
VE-PTP inhibits VE-cadherin phosphorylation	In vitro (CHO)	Transfection with VEGFR2, VE-cadherin and inducible inactivated VE-PTP	Mifepristone (to induce expression of inactivated VE-PTP)	VE-cadherin protein phosphorylation status and paracellular permeability (FITC) were reduced by induction of both WT and inactivated VE-PTP	Study shows that VE-PTP does not require its phosphatase activity to prevent VE-cadherin phosphorylation	Nawroth et al. (2002)
Tie2 stimulates VE-PTP-dependent dephosphorylation of VEGFR2	In vitro (HUVEC)	Endogenous pathway	Angtp1 + VEGF + VE-PTP siRNA	VEGF-induced increase in VEGFR2 protein phosphorylation status was reduced by co-stimulation with Angpt1. This effect was abolished by VE-PTP knockdown		Hayashi et al. (2013)
			VEGF + Tie2 siRNA	VEGF-induced VEGFR2 protein phosphorylation status was increased in Tie2 knockdown cells		
mDia sequesters Src and competes with VEGFR2	In vitro (Seminal Vesicle Epithelial Cells; SVEC)	Transfected with myc-active mDia1	VEGF + increasing levels of mDia1 activity	VEGF-stimulated VEGFR2 association with Src was reduced in a dose-dependent manner by mDia1, while mDia1 association with Src increased dose-dependently	5 min VEGF stimulation	Gavard et al. (2008)
Src phosphorylates FAK	In vitro (COS-7)	Transfected with WT FAK	Isolated c-Src	FAK protein phosphorylation status was increased on several activation sites upon exposure to Src		Calalb et al. (1995)

FAK activates Ras/ERK	In vivo (chick chorioallantoic membrane; CAM)	Transduction with FAK-related nonkinase (FRNK)	VEGF	VEGF-induced increase in activated Ras and ERK protein phosphorylation status were inhibited by FRNK		Hood et al. (2003)
Src activates Ras/ERK		Endogenous pathway	VEGF + pharmacological Src inhibitor PP1	VEGF-induced increase in Raf and ERK protein phosphorylation status was inhibited by PP1	Raf is located downstream of Ras	
